# Transcriptomic Changes Triggered by Hypoxia: Evidence for HIF-1α -Independent, [Na^+^]_i_/[K^+^]_i_-Mediated, Excitation-Transcription Coupling

**DOI:** 10.1371/journal.pone.0110597

**Published:** 2014-11-06

**Authors:** Svetlana V. Koltsova, Boris Shilov, Julia G. Birulina, Olga A. Akimova, Mounsif Haloui, Leonid V. Kapilevich, Svetlana V. Gusakova, Johanne Tremblay, Pavel Hamet, Sergei N. Orlov

**Affiliations:** 1 Department of Biology, Moscow State University, Moscow, Russia; 2 Department of Medicine, Centre de recherche, Centre hospitalier de l'Université de Montréal, Montreal, Quebec, Canada; 3 Department of Physiology, Pirogov Russian National Research Medical University, Moscow, Russia; 4 Department of Medical Biology, Siberian State Medical University, Tomsk, Russia; 5 Department of Physical Education, Tomsk State University, Tomsk, Russia; University of Pittsburgh School of Medicine, United States of America

## Abstract

This study examines the relative impact of canonical hypoxia-inducible factor-1alpha- (HIF-1α and Na^+^_i_/K^+^_i_-mediated signaling on transcriptomic changes evoked by hypoxia and glucose deprivation. Incubation of RASMC in ischemic conditions resulted in ∼3-fold elevation of [Na^+^]_i_ and 2-fold reduction of [K^+^]_i_. Using global gene expression profiling we found that Na^+^,K^+^-ATPase inhibition by ouabain or K^+^-free medium in rat aortic vascular smooth muscle cells (RASMC) led to the differential expression of dozens of genes whose altered expression was previously detected in cells subjected to hypoxia and ischemia/reperfusion. For further investigations, we selected *Cyp1a1, Fos, Atf3, Klf10, Ptgs2, Nr4a1, Per2* and *Hes1*, i.e. genes possessing the highest increments of expression under sustained Na^+^,K^+^-ATPase inhibition and whose implication in the pathogenesis of hypoxia was proved in previous studies. In ouabain-treated RASMC, low-Na^+^, high-K^+^ medium abolished amplification of the [Na^+^]_i_/[K^+^]_i_ ratio as well as the increased expression of all tested genes. In cells subjected to hypoxia and glucose deprivation, dissipation of the transmembrane gradient of Na^+^ and K^+^ completely eliminated increment of *Fos, Atf3, Ptgs2* and *Per2* mRNAs and sharply diminished augmentation expression of *Klf10, Edn1, Nr4a1* and *Hes1*. In contrast to low-Na^+^, high-K^+^ medium, RASMC transfection with *Hif-1a* siRNA attenuated increments of *Vegfa, Edn1, Klf10* and *Nr4a1* mRNAs triggered by hypoxia but did not impact *Fos, Atf3, Ptgs2* and *Per2* expression. Thus, our investigation demonstrates, for the first time, that Na^+^_i_/K^+^_i_-mediated, Hif-1α- -independent excitation-transcription coupling contributes to transcriptomic changes evoked in RASMC by hypoxia and glucose deprivation.

## Introduction

Hypoxia is characteristic of numerous pathologies, including inflammation [1], cancer [2], obesity [3], systemic and pulmonary hypertension [4]; [5], atherosclerosis [6] and kidney disease [7]. In 1986, Murry and colleagues reported that the size of myocardial infarcts, arising from 40-min occlusion of the circumplex artery, could be reduced by 75% if the myocardium had been subjected to so-called ischemic preconditioning, i.e., several short occlusions interspersed by periods of reperfusion [8]. Later on, the protective action of brief ischemia was documented in other tissues, including blood vessels [9]. Significantly, the prophylactic influence of ischemic preconditioning was at least partially blocked by inhibitors of RNA synthesis [10]; [11], suggesting a key role of profound transcriptomic changes documented in global gene expression profiling studies of ischemic tissues [12]–[18].

Hypoxia-inducible factor 1alpha (HIF-1α), considered to be a major oxygen sensor, regulates gene expression in ischemic tissues via interaction of HIF-1α/HIF-1β heterodimer with hypoxia response elements (HREs) in promoter/enhancer regions of the target gene's DNA. In normoxia, HIF-1α is hydroxylated by oxygen-dependent prolyl hydrolase that elicits its proteasomal degradation. In contrast, under hypoxic conditions, HIF-1α is translocated to the nucleus, where it forms HIF-1α/HIF-1β complex. The list of HIF-1-sensitive genes comprises *Hif-1α per se*, and others related to vasomotor control (nitric oxide synthase-2, adrenomedulin, endothelin-1), angiogenesis (vascular endothelial growth factor (*Vegf*) and its receptor *Flt1*), erythropoiesis and iron metabolism (erythropoietin, transferrin, transferrin receptor, ceruloplasmin), cell proliferation (*Igf1*, *Igfbp1, Tgfb*), energy metabolism (glucose transporters *Glut1-Glut3*, phosphoenolpyruvate carboxylase, lactate dehydrogenase A, aldose, phosphoglucokinase-1, -L and -C, endolase, tyrosine hydroxylase and plasminogen activator inhibitor-1) (for review see, [10]; [19]-[22].

Immediately after attenuation of oxygen partial pressure and delivery of cell fuels caused by cessation of blood flow, the concentration of ATP and other high-energy phosphate compounds falls, which, in turn, leads to declining ion pump activities, dissipation of electrochemical gradients of K^+^, Na^+^, Cl^-^ and Ca^2+^ and plasma membrane depolarization [23]. Numerous research teams reported that [Ca^2+^]_i_ elevation triggers cell damage via activation of Ca^2+^-sensitive isoforms of proteases, protein kinase C, mitogen-activated protein kinase, JNK and p38 as well as transcriptomic alterations evoked by Ca^2+^_i_-sensitive transcriptional elements, such as Ca^2+^-response elements (CRE), serum-response element (SRE) and activating protein-1 (AP-1) [24]. Post-ischemic reperfusion appears to induce further damage via mitochondrial Ca^2+^ overload and production of reactive oxygen species, including superoxide, hydroxyl, and nitric oxide radicals [25]. Recently, we noted, however, that in several types of mammalian cells Ca^2+^-depletion increased rather than decreased the number of transcripts whose differential expression was triggered by Na^+^,K^+^-ATPase inhibition [26]. These data motivate us to propose that, side-by-side with the above-listed signaling pathways, transcriptomic changes in ischemic tissues are evoked by excitation-transcription coupling via a novel Na^+^_i_/K^+^_i_-mediated, Ca^2+^_i_-independent mechanism. We designed the present study to examine this hypothesis.

## Results

### Effect of ouabain, K+-free medium and hypoxia on intracellular content of monovalent ions and ATP

**Figure 1:**
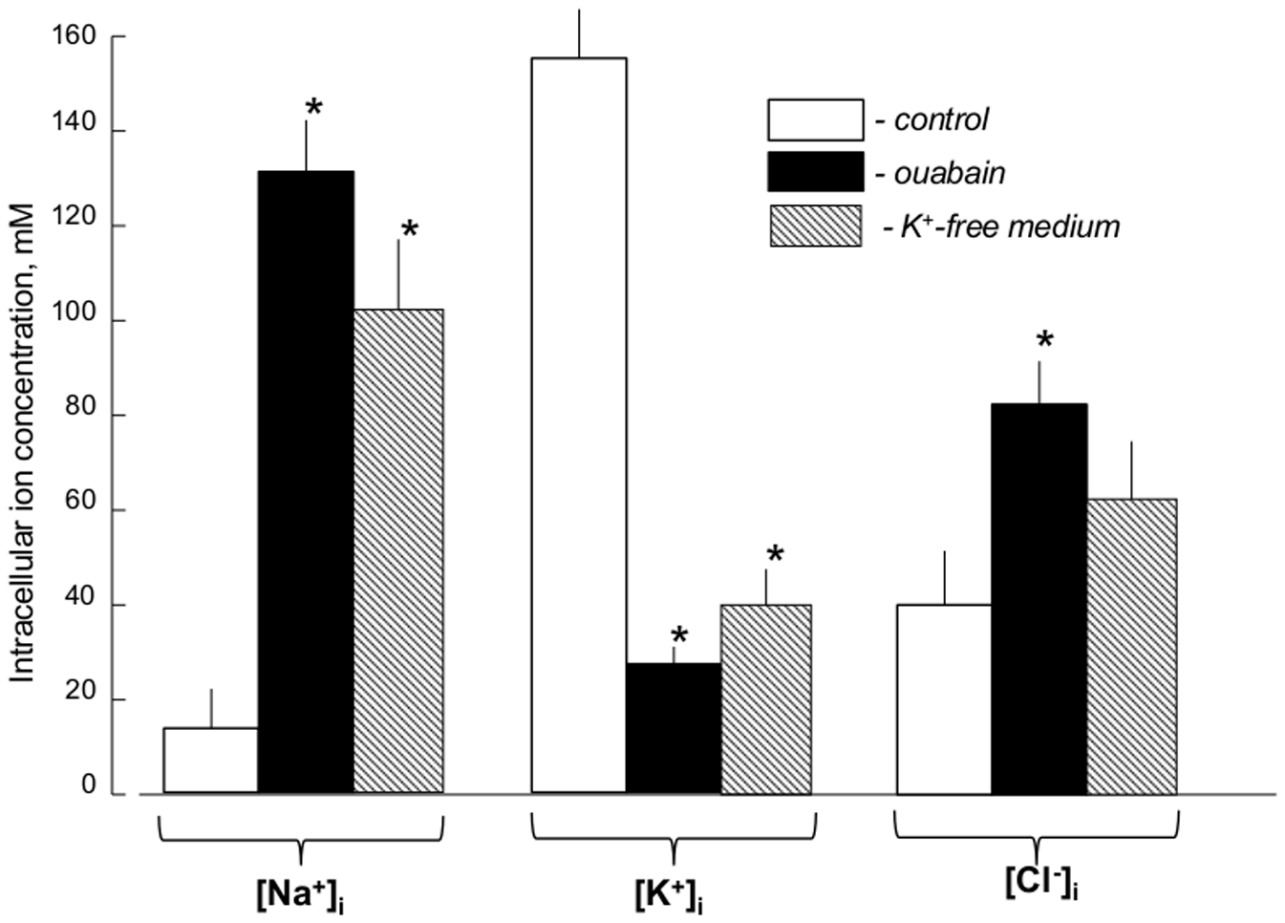
Effect of Na^+^,K^+^-ATPase inhibition on the intracellular content of monovalent ions. RASMC were incubated in control and K^+^-free medium or in the presence of 3 mM ouabain for 6 hr. Means ± S.E. from 3 independent experiments performed in quadruplicate are shown. *p<0.05 compared to controls.

Six-hr inhibition of Na^+^,K^+^-ATPase in RASMC by ouabain increased [Na^+^]_i_ from 15–20 to 130 mM and decreased [K^+^]_i_ from ∼150 to 25 mM (Fig. 1). Somewhat similar elevation of the [Na^+^]_i_/[K^+^]_i_ ratio was detected with 6 hr of Na^+^,K^+^-ATPase inhibition in K^+^-free medium. Dissipation of the transmembrane gradients of monovalent cations, triggered by ouabain and K^+^-free medium, was accompanied by elevation of [Cl^-^]_i_ from ∼40 to 80 and 60 mM, respectively (Fig. 1).

**Figure 2:**
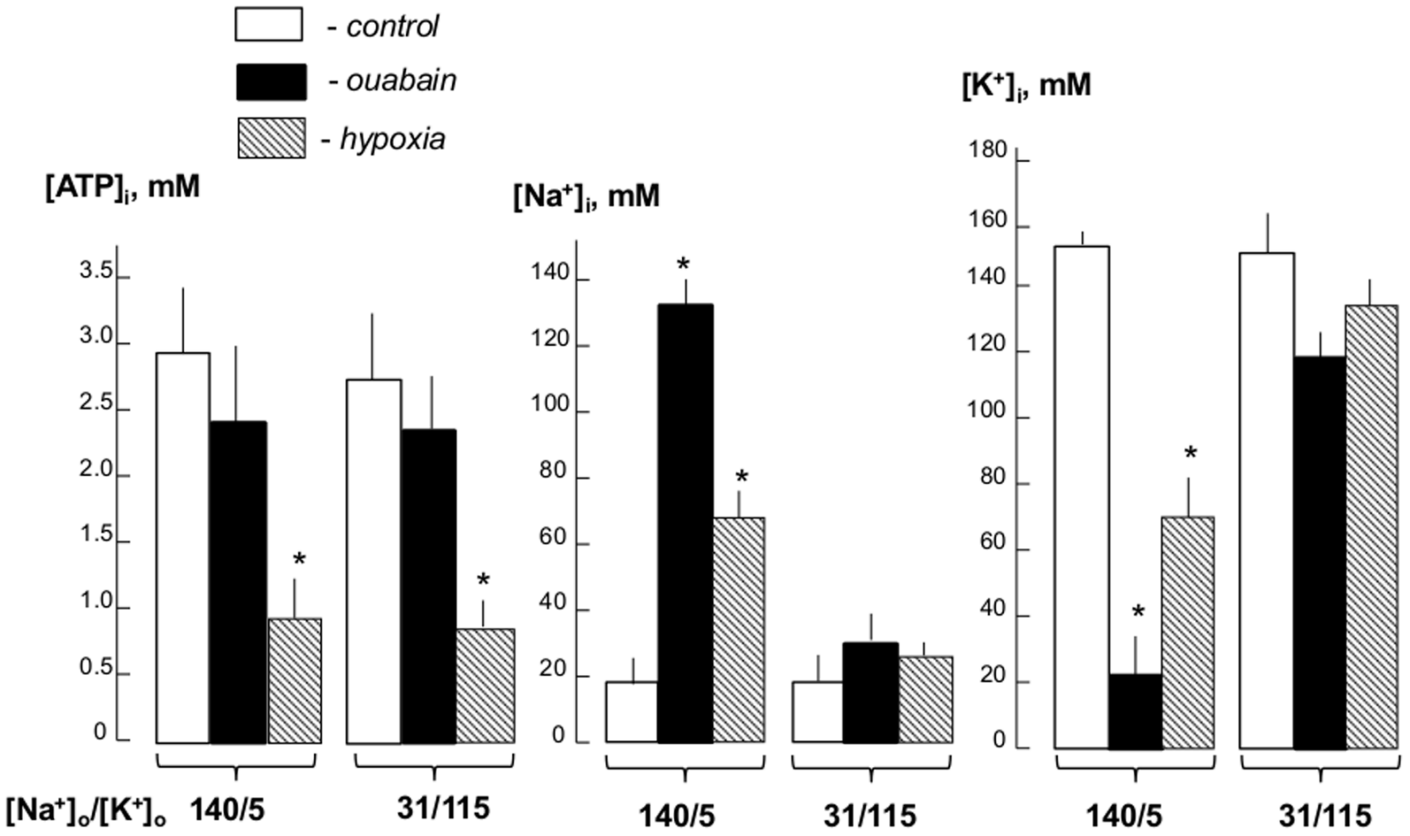
Effect of ouabain and hypoxia on intracellular Na^+^, K^+^ and ATP concentrations. RASMC were incubated for 24 hr under normal oxygen partial pressure (5% CO_2_/air - *control*) ±3 µM ouabain or exposure to hypoxia (5% CO_2_/95% N_2_)/glucose deprivation in normal high-Na^+^, low-K^+^ ([Na^+^]_o_/[K^+^]_o_ = 140/5) or in low-Na^+^, high-K^+^ DMEM-like medium ([Na^+^]_o_/[K^+^]_o_ = 131/115). Means ± S.E. from 3 independent experiments performed in quadruplicate are shown. *p<0.05 compared to the controls.

Figure 2 shows that 24-hr incubation of RASMC in hypoxia and glucose starvation decreased intracellular ATP content by ∼3-fold whereas ouabain attenuated this parameter by less than 20%. The actions of hypoxia and ouabain on ATP content were preserved in low-Na^+^, high-K^+^ medium. Treatment with ouabain resulted in almost 10-fold gain of [Na^+^]_i_ and virtually similar loss of [K^+^]_i_. In hypoxic conditions, [Na^+^]_i_ and [K^+^]_i_ were increased and decreased by 3- and 2-fold, respectively. As predicted, dissipation of the transmembrane gradients of monovalent cations in low-Na^+^, high-K^+^ medium almost completely abolished the actions of ouabain and hypoxia on the [Na^+^]_i_/]K^+^]_i_ ratio (Fig. 2). Viewed collectively, these results allowed us to hypothesize that transcriptomics changes triggered by hypoxia are at least partially caused by Na^+^_i_/K^+^_i_-mediated excitation-transcription coupling discovered in our recent studies [26]. Data considered below support this hypothesis.

### Identification and functional characterization of [Na^+^]_i_/]K^+^]_i_-sensitive transcriptome in RASMC

**Figure 3:**
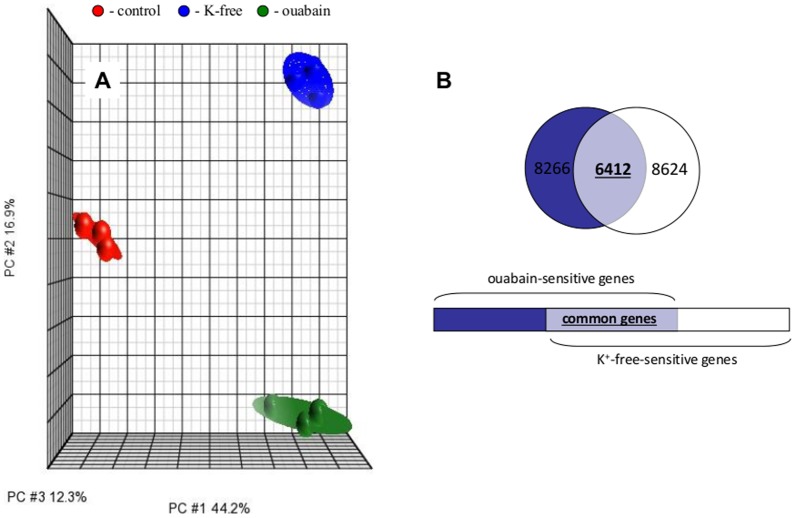
Effect of Na^+^,K^+^-ATPase inhibition on the RASMC transcriptome. Cells were incubated for 6 hr in control DMEM, K^+^-free DMEM or DMEM containing 3 mM ouabain. All experiments are repeated 3 times. **A**. PCA of transcriptomic changes. Ellipsoids highlight portioning of samples based on type of treatment. The principal components in 3-dimensional graphs (PC#1, PC#2 and PC#3) represent the variability of gene expression level within datasets. **B**. Comparative analysis of the impact of Na^+^,K^+^-ATPase inhibition by ouabain and K^+^-free medium on the RASMC transcriptome. The total number of genes whose expression is altered by ouabain and K^+^-free medium by more than 1.2-fold with p<0.05 is indicated; the number of genes affected by both stimuli appears in **bold**.

Affymetrix data from 3 independent experiments were normalized and analyzed by PCA [27]. Each point on PCA represents the gene expression profile of an individual sample. Samples that are near each other in the resulting 3-dimensional plot have a similar transcriptome while those that are further apart have dissimilar transcriptional profiles. This approach identified ouabain and K^+^-free medium as major sources of variability within datasets (Fig. 3A).

**Figure 4:**
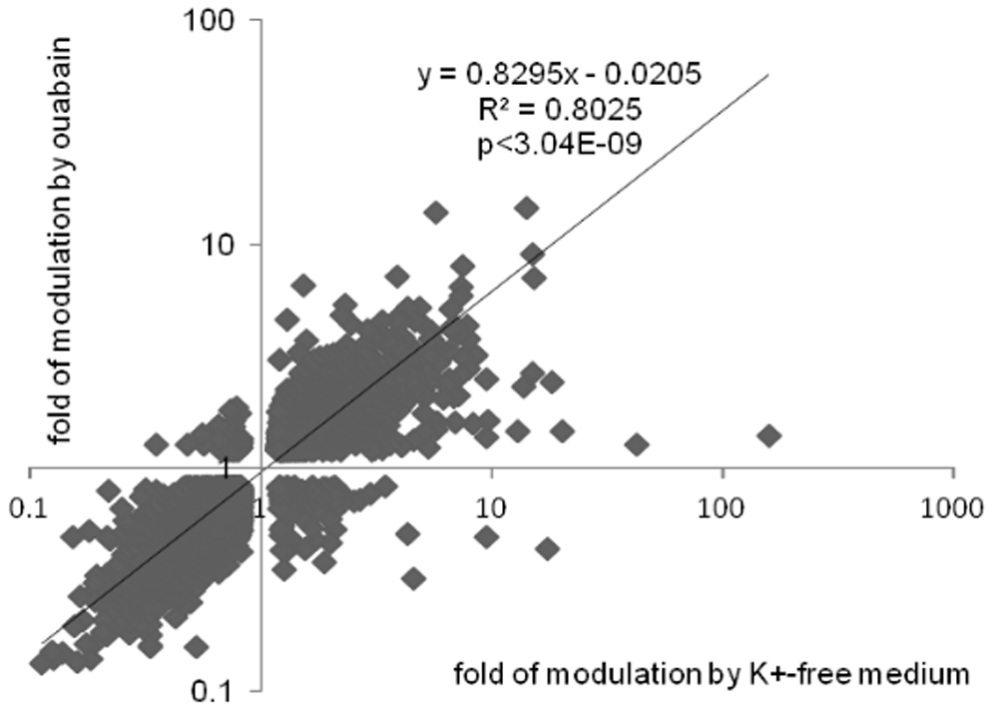
Correlation analysis of transcripts whose expression is altered by ouabain and K^+^-free medium in RVSMC by more than by 1.2-fold with p<0.05. The total number of transcripts subjected to analysis is shown in Figure 2B. Transcript expression in control cells was taken as 1.00. The fold change was determined as log transformed treatment/control expression ratio.

Figure 3B discloses that the number of differentially-expressed transcripts in RASMC treated for 6 hr with ouabain or K^+^-free medium totalled 8,266 and 8,264, respectively. Further analysis determined that the expression of 6,412 transcripts was affected by both stimuli (Fig. 3B). Significantly, we observed highly significant (p<4×10^−9^) and positive (R^2^>0.80) correlations between levels of differentially-expressed transcripts identified in the presence of ouabain and K^+^-free medium (Fig. 4). Because the gain of [Na^+^]_i_ and loss of [K^+^]_i_ in cells treated with ouabain or K^+^-free medium are similar (Fig. 1), the results strongly suggest that the changes in gene expression evoked by both stimuli occur in response to elevation of the [Na^+^]_i_/[K^+^]_i_ ratio rather than [Na^+^]_i_/[K^+^]_i_-independent events.

It should be noted that, together with increment of the [Na^+^]_i_/[K^+^]_i_ ratio, ouabain and K^+^-free medium may affect cells independently of suppression of Na^+^,K^+^-ATPase-mediated ion fluxes. Thus, recent studies revealed that ouabain triggered interaction of the Na^+^,K^+^-ATPase α-subunit with the membrane-associated nonreceptor tyrosine kinase Src, activation of Ras/Raf/ERK1,2, phosphatidyl inositol 3-kinase (PI(3)K), PI(3)K-dependent protein kinase B, phospholipase C, [Ca^2+^]_i_ oscillations and augmented production of ROS (for review, see [28]; [29]). On the other hand, transfer of highly K^+^-permeable cells to K^+^-free medium results in transient membrane hyperpolarization that has a tissue-specific impact on the activity of diverse voltage-sensitive, membrane-bound proteins [30]; [31] and the distribution of other permeable ions, including Cl^-^ (Fig. 1). Indeed, we noted that the expression of several genes detected, such as *Cxcl2, Cxcl5, Tnfs5, Lif* and *Vcam1*, is sharply increased in K^+^-free medium compared to ouabain-treated cells (Table 1). This considered, we focused our analysis on [Na^+^]_i_/[K^+^]_i_-sensitive genes whose expression in K^+^-free medium and in the presence of ouabain was less than 2-fold different. [Na^+^]_i_/[K^+^]_i_-sensitive genes, whose expression was increased or decreased by more that 3-fold, are listed in Tables 2 and 3, respectively.

**Table 1:** Genes whose expression was increased in RASMC subjected to Na^+^,K^+^-ATPase inhibition.

Gene symbol//gene title (ref #) – functional categories	K^+^-free medium		ouabain	
	Fold change	p value	Fold change	p value
Gdf15//growth differentiation factor 15 (D) - D	15.17	1.88E-06	7.44	6.03E-06
**Ereg//epiregulin (1) – D**	**15.14**	**1.59E-06**	**9.63**	**4.10E-06**
**Cyp1a1//cytochrome P450, family 1, subfamily a (15) - O**	**14.22**	**1.59E-06**	**15.58**	**2.95E-06**
**Fos//FBJ osteosarcoma oncogene (427) – T**	**7.91**	**2.23E-05**	**3.95**	**1.23E-04**
**Atf3//activating transcription factor (27) – T**	**7.83**	**2.85E-05**	**4.53**	**1.07E-04**
Slc9a3//solute carrier family 9 (sodium/hydrogen exchang – O	7.52	2.13E-06	3.25	1.22E-05
Nppc//natriuretic peptide C – O	7.42	6.35E-06	6.80	9.53E-06
Bhlhe41//basic helix-loop-helix family, member e41 – T	7.42	6.59E-06	6.23	1.13E-05
**Klf10//Kruppel-like factor 10 (2) – T**	**7.16**	**4.32E-06**	**4.09**	**1.38E-05**
**Trib1//Tribbles homolog 1 (Drosophila) (2) – D**	**7.09**	**1.50E-06**	**4.47**	**4.54E-06**
**Il6r//interleukin 6 receptor (1) – I**	**6.71**	**9.08E-07**	**4.37**	**3.17E-06**
*Ets2//v-ets erythroblastosis virus E26 oncogene homolog 2– T*	*6.68*	*4.93E-06*	*5.35*	*9.09E-06*
Fam43a//family with sequence similarity 43, member A – U	6.56	6.89E-06	3.45	3.34E-05
Arrdc4//arrestin domain containing 4– U	5.89	1.70E-06	3.06	7.44E-06
Tppp//tubulin polymerization promoting protein – D	5.89	1.11E-05	3.58	4.18E-05
**Ptgs2//prostaglandin-endoperoxide synthase 2 (588) – I**	**5.68**	**1.44E-06**	**3.71**	**4.45E-06**
*F3//coagulation factor III (thromboplastin, tissue factor I*	*5.64*	*1.62E-06*	*3.39*	*5.50E-06*
Enc1//ectodermal-neural cortex – D	5.61	9.08E-07	3.85	2.57E-06
*Mmp28//matrix metallopeptidase 28– I*	*5.47*	*3.64E-06*	*4.24*	*7.44E-06*
**Epha2//Eph receptor A2 (3) – D**	**5.46**	**2.73E-06**	**3.32**	**8.72E-06**
RGD1307396//similar to RIKEN cDNA 6330406I15 – U	5.39	2.12E-06	3.94	5.19E-06
Slc25a25//solute carrier family 25 (mitochondrial Pi carrier) – O	5.04	2.66E-06	2.85	1.12E-05
**Nr4a1//nuclear receptor subfamily 4, group A (14) – T**	**4.99**	**9.44E-06**	**4.67**	**1.33E-05**
**Per2//period homolog 2 (Drosophila) (6) – O**	**4.88**	**7.48E-06**	**5.47**	**8.33E-06**
Zbtb2//zinc finger and BTB domain containing 2– T	4.68	2.63E-06	3.09	8.01E-06
*Rab32//RAB32, member RAS oncogene family – T*	*4.68*	*1.49E-06*	*2.51*	*7.26E-06*
*Isg20//interferon stimulated exonuclease gene 20– I*	*4.67*	*2.44E-06*	*4.71*	*4.06E-06*
**Hbegf//heparin-binding EGF-like growth factor (4) – D**	**4.65**	**2.60E-06**	**2.69**	**1.10E-05**
*Pde4b//phosphodiesterase 4B, cAMP-specific – O*	*4.52*	*3.64E-06*	*2.43*	*2.15E-05*
Csrnp1//cysteine-serine-rich nuclear protein 1–D	4.52	3.63E-06	3.52	7.73E-06
PVR//poliovirus receptor - D	4.50	3.47E-06	3.58	7.26E-06
**Hes1//hairy and enhancer of split 1 (Drosophila) (34) – T**	**4.47**	**5.68E-06**	**2.94**	**1.93E-05**
**Bcl6//B-cell CLL/lymphoma 6 (1) –D**	**4.45**	**2.12E-06**	**2.90**	**7.26E-06**
*Ier5//immediate early response 5-T*	*4.45*	*5.59E-06*	*2.76*	*2.26E-05*
**Sgk1//serum/glucocorticoid regulated kinase 1 (14) - O**	**4.42**	**3.96E-06**	**3.64**	**7.69E-06**
**Plk3//polo-like kinase 3 (20) -D**	**4.41**	**1.03E-05**	**3.13**	**3.05E-05**
*Klf5//Kruppel-like factor 5-T*	*4.39*	*1.17E-05*	*3.43*	*2.64E-05*
Mthfd2l//methylenetetrahydrofolate dehydrogenase – O	4.38	9.85E-06	2.49	6.04E-05
**Areg//amphiregulin (3) – D**	**4.37**	**1.00E-05**	**3.26**	**2.51E-05**
RGD1563891//similar to core 2 beta-1,6-N-acetylglucosam – U	4.36	5.54E-05	3.52	1.17E-04
**Fst//follistatin (9) – D**	**4.35**	**6.59E-06**	**5.50**	**6.34E-06**
*Fosl1//fos-like antigen 1-T*	*4.27*	*1.20E-05*	*3.41*	*2.59E-05*
**Ier2//immediate early response 2 (1) – T**	**4.24**	**2.62E-05**	**1.99**	**6.15E-04**
Baiap2//BAI1-associated protein 2-D	4.14	9.08E-07	3.98	2.57E-06
Rassf9//Ras association (RalGDS/AF-6) domain family – D	4.12	2.41E-06	2.86	7.26E-06
Chka//choline kinase alpha – O	4.10	1.82E-06	4.71	2.95E-06
*Irf2bpl//interferon regulatory factor 2-binding protein-like – I*	*4.10*	*3.37E-05*	*3.27*	*7.69E-05*
Mum1l1//melanoma-associated antigen (mutated) 1-like 1–I	4.00	4.60E-06	2.69	1.53E-05
**Nr4a3//nuclear receptor subfamily 4, group A, mem 3 (5) – T**	**3.99**	**1.00E-05**	**4.13**	**1.19E-05**
Tas2r135//taste receptor, type 2, member 135–O	3.94	3.86E-05	3.56	6.11E-05
Errfi1//ERBB receptor feedback inhibitor 1–D	3.93	4.30E-06	2.82	1.19E-05
Skil//SKI-like oncogene - T	3.92	9.08E-07	2.13	6.03E-06
Zswim4//zinc finger, SWIM-type containing 4–T	3.92	2.09E-06	2.31	1.02E-05
**Dusp10//dual specificity phosphatase 10 (3)-D**	**3.92**	**8.95E-06**	**2.09**	**8.82E-05**
**Has2//hyaluronan synthase 2 (3) –I**	**3.91**	**9.85E-06**	**7.60**	**4.67E-06**
**Txnip//thioredoxin interacting protein (9) – I**	**3.88**	**1.82E-06**	**2.31**	**8.58E-06**
**Mdm2//Mdm2 p53 binding protein homolog (mouse) (22) – T**	**3.85**	**9.08E-07**	**2.98**	**2.95E-06**
Fam171b//family with sequence similarity 171, member B – U	3.84	3.05E-06	4.82	3.26E-06
Stk17b//serine/threonine kinase 17b –O	3.76	3.65E-06	4.00	4.67E-06
*Zfp36//zinc finger protein 36–T*	*3.70*	*6.56E-05*	*2.15*	*7.25E-04*
Rab20//RAB20, member RAS oncogene family –I	3.66	4.53E-06	3.90	5.52E-06
Pnrc1//proline-rich nuclear receptor coactivator 1–T	3.66	2.19E-07	3.10	7.72E-07
**Abcb1b//ATP-binding cassette, subfamily B, mem 1B (3) – O**	**3.65**	**9.08E-07**	**2.72**	**3.17E-06**
**Smad3//SMAD family member 3 (20) –T**	**3.64**	**4.27E-06**	**2.78**	**1.04E-05**
RGD1306119//similar to transcriptional regulating prot 132 – T	3.59	1.13E-05	1.80	2.42E-04
**Htr2a//5-hydroxytryptamine (serotonin) receptor 2A (1) – O**	**3.58**	**9.08E-07**	**3.10**	**2.78E-06**
**Jun//jun proto-oncogene (799) –T**	**3.57**	**1.77E-06**	**2.76**	**4.54E-06**
Zfat//zinc finger and AT hook domain containing – T	3.57	4.59E-06	3.01	8.69E-06
**Dusp6//dual specificity phosphatase 6 (2) -O**	**3.56**	**4.79E-06**	**2.42**	**1.73E-05**
Ier5l//immediate early response 5-like T	3.55	4.98E-06	2.71	1.29E-05
**Bmp2//bone morphogenetic protein 2 (13) –D**	**3.52**	**3.96E-05**	**3.69**	**4.14E-05**
Gnat1//guanine nucleotide-binding protein, alpha t –O	3.50	2.44E-06	3.86	3.26E-06
RGD1305254//similar to transmembrane protein 2–U	3.48	3.31E-05	2.14	2.69E-04
Alkbh//alkB, alkylation repair homolog (E. coli) –D	3.45	2.26E-06	2.44	7.40E-06
**Klf4//Kruppel-like factor 4 (gut) (5) –T**	**3.44**	**1.54E-05**	**2.49**	**5.57E-05**
Hapln3//hyaluronan and proteoglycan link protein 3 – O	3.44	2.58E-05	1.95	3.45E-04
*Mafk//v-maf musculoaponeurotic fibrosarcoma onco hom K–T*	*3.42*	*8.48E-06*	*2.75*	*1.91E-05*
*Maff//v-maf musculoaponeurotic fibrosarcoma onco hom F-T*	*3.42*	*2.09E-05*	*2.55*	*6.83E-05*
**Dusp16//dual specificity phosphatase 16 (2) –O**	**3.39**	**1.94E-05**	**2.23**	**1.10E-04**
Gprc5a//G protein-coupled receptor, family C, gr 5, mem A–O	3.38	3.26E-05	4.73	1.74E-05
Lgr5//leucine-rich repeat contain G protein coupled recept –O	3.32	1.11E-05	4.12	8.86E-06
**Per1//period homolog 1 (Drosophila) (4) –O**	**3.32**	**5.54E-06**	**4.09**	**5.06E-06**
**Cd80//Cd80 molecule (28) –I**	**3.30**	**6.40E-06**	**2.10**	**3.72E-05**
**Mybl1//myeloblastosis oncogene-like 1 (1) –T**	**3.29**	**1.86E-05**	**2.03**	**1.55E-04**
**Pim1//pim-1 oncogene (5) –D**	**3.27**	**6.86E-06**	**1.84**	**8.03E-05**
Osmr//oncostatin M receptor –I	3.25	2.76E-05	2.00	2.57E-04
**Smad7//SMAD family member 7 (6) –D**	**3.23**	**4.22E-06**	**2.05**	**2.23E-05**
**Cpeb4//cytoplasmic polyadenyl element bind prot 4 (1) –T**	**3.20**	**4.79E-06**	**3.01**	**7.42E-06**
**Aen//apoptosis-enhancing nuclease (5) D**	**3.17**	**5.96E-06**	**3.10**	**8.39E-06**
**Nr4a2//nuclear receptor subfamily 4, group A, mem 2 (4) – T**	**3.16**	**1.56E-05**	**3.99**	**1.15E-05**
**Tnfrsf1b//tumor necrosis factor receptor superfamily –D**	**3.15**	**2.67E-05**	**1.99**	**2.31E-04**
Lhfpl2//lipoma HMGIC fusion partner-like 2–O	3.10	4.60E-06	1.76	5.10E-05
Rap2b//RAP2B, member of RAS oncogene family –O	3.07	1.00E-05	2.34	3.03E-05
Lmbrd2//LMBR1 domain containing 2–U	3.07	9.85E-06	2.70	1.77E-05
Cdc42ep1//CDC42 effector protein (Rho GTPase binding) 1 – D	3.06	2.92E-05	2.80	4.71E-05
Kdm6b//lysine (K)-specific demethylase 6B –T	3.04	7.50E-06	2.04	3.94E-05
RGD1310794//similar to RIKEN cDNA C030048B08 - D	3.04	2.47E-04	4.01	1.07E-04
*Sertad2//SERTA domain containing 2-T*	*3.03*	*1.40E-05*	*2.55*	*3.05E-05*

RASMC were treated with 3 mM ouabain or K^+^-free medium for 6 hr. Listed are assigned genes whose expression was increased in K^+^-free medium by more than 3-fold and was different by less than 2-fold in the presence of ouabain compared to K^+^-free medium. GeneChip expression analysis was performed as described in the Methods section. mRNA content in control cells was taken as 1.00. Genes whose expression is altered in ischemia/hypoxia are shown **in bold**. Appearing in parentheses are numbers of citations in PubMed. Listed *in italics* are genes whose differential expression was detected in ischemic tissue by whole genome microarray-based analysis [12]–[18]. Data on gene function from GeneCards database (www.genecards.org) were used for identification of gene function. Functional categories: T– regulators of transcription/translation, RNA processing and degradation; D– regulators of cell adhesion, migration, proliferation, differentiation and death; I– inflammation and immune responses; O– others; U– unknown function.

**Table 2:** Genes whose expression was decreased in RASMC subjected to Na^+^,K^+^-ATPase inhibition.

Gene symbol//gene title (ref #) – functional categories	K^+^-free medium		ouabain	
	Fold change	p value	Fold change	p value
Syt17//synaptotagmin XVII -O	−3.01	1.19E-05	−1.81	1.35E-04
Phyh//phytanoyl-CoA 2-hydroxylase –O	−3.02	4.62E-05	−2.84	6.80E-05
Ttc21b//tetratricopeptide repeat domain 21B –O	−3.03	4.28E-05	−4.06	2.24E-05
Parp2//poly (ADP-ribose) polymerase 2–T	−3.03	2.91E-05	−3.94	1.77E-05
RGD1309534//similar to RIKEN cDNA 4931406C07 –U	−3.03	2.51E-05	−2.12	1.31E-04
Mus81//MUS81 endonuclease homolog (S. cerevisiae) – T	−3.03	8.76E-06	−2.52	1.88E-05
Lactb2//lactamase, beta 2–O	−3.04	2.89E-05	−3.41	2.48E-05
Fbxw17//F-box and WD-40 domain protein 17-U	−3.05	3.50E-05	−3.61	2.57E-05
**Gnb5//guanine nucleotide binding prot beta 5 (1) – O**	**−3.05**	**1.03E-05**	**−2.63**	**2.03E-05**
Mrpl32//mitochondrial ribosomal protein L32 –O	−3.06	5.76E-05	−1.83	8.71E-04
MGC94199//similar to RIKEN cDNA 2610301B20; -U	−3.06	2.15E-04	−3.68	1.26E-04
Zscan12//zinc finger and SCAN domain containing 12–T	−3.07	2.82E-05	−3.37	2.57E-05
Top3b//topoisomerase (DNA) III beta –T	−3.07	9.85E-06	−3.90	7.40E-06
*Zfp259//zinc finger protein 25 –T*	−*3.08*	*3.32E-06*	−*1.61*	*5.70E-05*
Myo19//myosin XIX –O	−3.09	1.64E-05	−2.24	6.71E-05
**Prkag1//protein kinase, AMP-activat, gamma 1 (1) –O**	**−3.09**	**4.04E-06**	**−1.67**	**6.07E-05**
Gtpbp5//GTP-binding protein 5–T	−3.10	2.84E-05	−2.83	4.59E-05
Dcaf4//DDB1 and CUL4 associated factor 4–O	−3.10	1.20E-05	−2.59	2.64E-05
LOC687284//similar to excision repair cross-compl –U	−3.10	7.20E-06	−3.34	8.30E-06
*Gdf10//growth differentiation factor 10–D*	−*3.11*	*9.18E-05*	−*2.69*	*1.83E-04*
Tmem184c//transmembrane protein 184C –D	−3.11	3.45E-06	−2.02	1.69E-05
Deadc1//deaminase domain containing 1–O	−3.11	4.60E-05	−3.13	5.42E-05
RGD1311422//similar to CG8841-PA –U	−3.12	9.85E-06	−2.60	2.07E-05
**Parp1//poly (ADP-ribose) polymerase 1 (46) –D**	**−3.13**	**1.87E-06**	**−2.75**	**4.29E-06**
Rtel1//regulator of telomere elongation helicase 1 –D	−3.13	8.33E-06	−2.29	2.80E-05
Dhcr7//7-dehydrocholesterol reductase –O	−3.14	3.83E-05	−4.86	1.54E-05
Gatsl2//GATS protein-like 2–T	−3.14	3.51E-05	−1.97	3.31E-04
Glb1l2//galactosidase, beta 1-like 2–O	−3.14	2.49E-05	−3.60	2.09E-05
RGD1563798//similar to BC040823 protein –U	−3.14	2.24E-05	−2.88	3.62E-05
Gbas//glioblastoma amplified sequence –O	−3.15	2.23E-06	−2.92	4.31E-06
*Cybasc3//cytochrome b, ascorbate dependent 3–O*	−*3.15*	*4.82E-06*	−*3.72*	*4.97E-06*
Fn3krp//fructosamine-3-kinase-related protein –O	−3.16	2.94E-06	−3.92	3.24E-06
Mrpl34//mitochondrial ribosomal protein L34 –T	−3.17	3.78E-04	−2.27	1.88E-03
Nsun6//NOP2/Sun domain family, member 6–U	−3.17	1.49E-05	−1.99	1.19E-04
Zdhhc12//zinc finger, DHHC-type containing 12–T	−3.17	5.52E-05	−4.22	2.93E-05
Rabl5//RAB, member RAS oncogene family-like 5–U	−3.18	5.97E-05	−2.89	9.60E-05
*Aacs//acetoacetyl-CoA synthetase –O*	−*3.18*	*5.96E-06*	−*2.42*	*1.68E-05*
Xylb//xylulokinase homolog (H. influenzae) –U	−3.19	1.70E-06	−2.51	4.54E-06
Lhfp//lipoma HMGIC fusion partner –U	−3.19	4.12E-06	−2.65	8.66E-06
**Prkag2//protein kinase, AMP-activat gamma 2 (4) –O**	**−3.20**	**3.79E-06**	**−2.61**	**8.39E-06**
Six2//SIX homeobox 2 (1) –T	−3.20	3.23E-06	−2.19	1.19E-05
Uri1//URI1, prefoldin-like chaperone –T	−3.21	4.03E-06	−3.34	5.07E-06
Aarsd1//alanyl-tRNA synthetase domain containing 1–T	−3.21	1.80E-05	−3.10	2.46E-05
*Pik3r2//phosphoinositide-3-kinase, regulatory subunit 2- O*	−*3.22*	*1.03E-05*	−*2.96*	*1.62E-05*
Mkrn2//makorin, ring finger protein, 2–O	−3.22	6.43E-05	−2.84	1.16E-04
Map9//microtubule-associated protein 9–D	−3.25	1.70E-05	−2.97	2.74E-05
Dnajc2//DnaJ (Hsp40) homolog, subfamily C, memb 2–T	−3.25	1.73E-05	−2.29	7.41E-05
Asb13//ankyrin repeat and SOCS box-containing 13–O	−3.26	3.47E-06	−2.65	7.73E-06
RGD1561270//similar to zinc finger protein 248–T	−3.26	2.32E-05	−3.71	2.04E-05
Hebp2//heme-binding protein 2–O	−3.26	6.69E-05	−2.54	2.00E-04
Cers4//ceramide synthase 4–O	−3.28	5.44E-05	−2.94	9.07E-05
MGC108823//similar to interferon-inducible GTPase –U	−3.28	1.20E-04	−3.17	1.52E-04
*Bmyc//brain expressed myelocytomatosis oncogene –T*	−*3.28*	*2.90E-05*	−*3.01*	*4.61E-05*
Pitpna//phosphatidylinositol transfer protein, alpha –O	−3.28	9.08E-07	−2.84	2.91E-06
Npepl1//aminopeptidase-like 1 – O	−3.29	7.69E-06	−3.32	9.65E-06
**Mtrr//5-methyltetrahydrofolate-homocystein (2) –O**	**−3.29**	**1.07E-05**	**−3.02**	**1.73E-05**
Akap6//A kinase (PRKA) anchor protein 6–O	−3.30	3.74E-05	−3.60	3.49E-05
Ttc8//tetratricopeptide repeat domain 8–D	−3.30	5.05E-06	−3.72	5.61E-06
Dgcr8//DiGeorge syndrome critical region gene 8–T	−3.30	2.90E-06	−1.70	3.90E-05
Mgat4b//mannosyl (alpha-1,3-)-glycoprotein beta –O	−3.30	3.01E-05	−3.84	2.40E-05
LOC687284//similar to excision repair cross-complem –U	−3.32	2.05E-05	−3.60	2.05E-05
Rpusd1//RNA pseudouridylate synthase domain-cont –T	−3.32	1.85E-05	−2.74	4.20E-05
LOC500420//similar to CG12279-PA –U	−3.33	2.38E-04	−5.21	7.30E-05
Stxbp2//syntaxin-binding protein 2–O	−3.33	9.86E-06	−4.00	8.49E-06
Mis18bp1//MIS18-binding protein 1–U	−3.34	6.29E-06	−1.76	1.02E-04
**Nol3//nucleolar protein 3, apoptosis repressor (16) – D**	**−3.34**	**4.84E-05**	**−2.16**	**3.44E-04**
Lst8//MTOR-associated protein, LST8 homolog – T	–3.34	1.25E-05	–2.80	2.66E-05
Bbs9//Bardet-Biedl syndrome 9–O	–3.35	6.12E-06	–2.70	1.38E-05
**Apip//APAF1-interacting protein (1) –D**	**–3.35**	**9.85E-06**	**–3.22**	**1.35E-05**
**Rabif//RAB-interacting factor (1) –O**	**–3.35**	**9.08E-07**	**–1.71**	**7.26E-06**
*Vsnl1//visinin-like 1–O*	*−3.36*	*1.00E-04*	*−3.19*	*1.37E-04*
Fig4//FIG4 homolog, SAC1 lipid phosphatase domain –O	*−*3.37	9.33E-06	*−*3.14	1.37E-05
Tmem209//transmembrane protein 209–U	*−*3.37	1.96E-05	*−*4.16	1.46E-05
LOC685574//zinc finger protein 334-T	*−*3.38	3.36E-05	*−*3.18	4.91E-05
Arhgef25//Rho guanine nucleotide exchange factor 25–D	*−*3.40	5.05E-06	*−*3.13	8.33E-06
Vamp4//vesicle-associated membrane protein 4–O	*−*3.40	1.52E-04	*−*2.58	5.08E-04
Pold2//polymerase (DNA directed), delta 2, regulat sub –D	*−*3.40	2.88E-05	*−*2.06	2.70E-04
Ssbp1//single-stranded DNA-binding protein 1–O	*−*3.40	6.62E-06	*−*3.45	8.57E-06
Slc35d2//solute carrier family 35, member D2–O	*−*3.40	6.67E-06	*−*3.32	9.24E-06
LOC683626//similar to limb-bud and heart –U	*−*3.41	1.30E-05	*−*1.84	2.13E-04
Lrrk1//leucine-rich repeat kinase 1–O	*−*3.41	4.33E-05	*−*3.31	5.60E-05
RGD1309362//similar to interferon-inducible GTPase –O	*−*3.41	1.21E-04	*−*3.64	1.10E-04
Acat3//acetyl-coenzyme A acetyltransferase 3–O	*−*3.41	4.09E-06	*−*2.35	1.42E-05
Pter//phosphotriesterase related –O	*−*3.42	8.74E-06	*−*2.31	3.69E-05
RragB//Ras-related GTP-binding B–D	*−*3.43	1.93E-04	*−*2.56	7.02E-04
**Pdcd4//programmed cell death 4 (5) –D**	***−*** **3.43**	**1.28E-05**	***−*** **1.74**	**3.18E-04**
Aaas//achalasia, adrenocortical insufficiency, alacrimia – O	*−*3.44	3.64E-06	*−*2.53	1.03E-05
Bphl//biphenyl hydrolase-like (serine hydrolase) O	*−*3.45	1.38E-05	*−*3.96	1.28E-05
Zfp68//zinc finger protein 68–T	*−*3.45	1.17E-03	*−*3.40	1.31E-03
Stambp//Stam-binding protein –I	*−*3.45	2.04E-05	*−*4.59	1.33E-05
Ptcd2//pentatricopeptide repeat domain 2–U	*−*3.46	2.03E-04	*−*4.01	1.41E-04
Aurkb//aurora kinase B–D	*−*3.47	8.48E-06	*−*1.84	1.24E-04
Mvd//mevalonate (diphospho) decarboxylase –O	*−*3.49	5.26E-05	*−*2.07	5.61E-04
Ssh3//slingshot homolog 3 (Drosophila) –O	*−*3.50	1.82E-06	*−*3.39	3.26E-06
Zfp110//zinc finger protein 110–T	*−*3.50	1.03E-05	*−*3.33	1.46E-05
*Hscb//HscB iron-sulfur cluster co-chaperone homolog – O*	*−3.50*	*2.66E-05*	*−3.00*	*5.16E-05*
*Tfip11//tuftelin-interacting protein 11–D*	*−3.51*	*5.98E-06*	*−4.67*	*5.00E-06*
Thtpa//thiamine triphosphatase/-O	*−*3.52	1.26E-05	*−*3.27	1.96E-05
LOC100362431//tetratricopeptide repeat domain 30B –U	*−*3.52	2.88E-05	*−*3.00	5.70E-05
RGD1564300//similar to phosphoseryl-tRNA kinase –U	*−*3.53	2.95E-05	*−*3.91	2.74E-05
Lrrc8e//leucine-rich repeat containing 8 family, memb E – D	*−*3.53	3.20E-04	*−*4.51	1.63E-04
Zfp39//zinc finger protein 39–T	*−*3.54	1.22E-04	*−*3.72	1.18E-04
Fbxl4//F-box and leucine-rich repeat protein 4–D	*−*3.55	1.87E-06	*−*3.72	3.17E-06
*Mpped2//metallophosphoesterase domain containing 2 – O*	*−3.55*	*2.65E-05*	*−3.28*	*4.02E-05*
Trak2//trafficking protein, kinesin binding 2–O	*−*3.55	8.54E-06	*−*2.34	3.83E-05
Ecsit//ECSIT homolog (Drosophila) –O	*−*3.55	1.18E-05	*−*3.75	1.34E-05
Tdp1//tyrosyl-DNA phosphodiesterase 1–D	*−*3.55	9.58E-06	*−*4.83	7.04E-06
*Pik3c2b//phosphoinositide-3-kinase, class 2, beta-O*	*−3.56*	*1.12E-05*	*−3.20*	*1.86E-05*
Nexn//nexilin (F actin binding protein) –D	*−*3.59	8.85E-05	*−*2.98	1.92E-04
**Impa2//inositol (myo)-1(or 4)-monophosphat 2 (1) - O**	***−*** **3.60**	**7.30E-05**	***−*** **2.24**	**5.86E-04**
Oasl2//2'-5' oligoadenylate synthetase-like 2-T	*−*3.60	4.54E-05	*−*4.51	3.03E-05
**Stk25//serine/threonine kinase 25 (1) –O**	***−*** **3.60**	**3.32E-06**	***−*** **2.97**	**6.95E-06**
Cnpy2//canopy 2 homolog (zebrafish) –D	*−*3.61	1.13E-05	*−*3.23	1.91E-05
Mrvi1//murine retrovirus integration site 1 homolog – U	*−*3.62	3.34E-06	*−*4.17	4.06E-06
Mrpl40//mitochondrial ribosomal protein L40 –T	*−*3.62	2.39E-05	*−*3.43	3.39E-05
Plin3//perilipin 3//9q11//316130 –O	*−*3.63	8.44E-06	*−*2.34	4.00E-05
Fam118a//family with sequence similarity 118, mem A –U	*−*3.64	5.05E-06	*−*4.01	6.02E-06
*Mmd//monocyte to macrophage differentiation-ass – U*	*−3.65*	*1.43E-05*	*−2.83*	*3.77E-05*
Hddc2//HD domain-containing 2 – U	*−*3.66	1.49E-05	*−*3.68	1.88E-05
Zfp386//zinc finger protein 386 (Kruppel-like) – T	*−*3.67	1.00E-04	*−*3.02	2.20E-04
Ficd//FIC domain-containing –O	*−*3.67	7.72E-06	*−*3.04	1.56E-05
Wdr11//WD repeat domain 11–T	*−*3.68	2.74E-05	*−*5.73	1.32E-05
Xrcc6//X-ray repair complement defective repair – D	*−*3.68	1.18E-05	*−*2.37	6.06E-05
Stx17//syntaxin 17–D	*−*3.68	1.03E-05	*−*5.02	7.40E-06
LOC100362548//rCG52086-like – U	*−*3.70	4.60E-06	*−*2.04	3.71E-05
Nipsnap3b//nipsnap homolog 3B (C. elegans) –O	*−*3.71	1.64E-05	*−*1.95	2.63E-04
Cad//carbamoyl-phosphate synthetase 2 –O	*−*3.72	1.09E-05	*−*2.72	3.33E-05
Gemin4//gem (nuclear organelle) associated protein 4 – T	*−*3.73	3.95E-05	*−*4.65	2.74E-05
Nagk//N-acetylglucosamine kinase –O	*−*3.73	4.98E-06	*−*4.28	5.47E-06
Tamm41//TAM41, mitochondrial translocator assembly – O	*−*3.73	7.27E-06	*−*2.99	1.61E-05
Msto1//misato homolog 1 (Drosophila) –O	*−*3.74	1.23E-05	*−*3.57	1.77E-05
**Mutyh//mutY homolog (E. coli) (3) –D**	***−*** **3.76**	**9.85E-06**	***−*** **3.14**	**1.91E-05**
**Stat2//signal transducer, activator of transcrip 2 (3) – T**	***−*** **3.76**	**1.50E-06**	***−*** **4.33**	**2.78E-06**
Gcs1//glucosidase 1–O	*−*3.81	3.65E-05	*−*2.03	5.66E-04
Ccdc36//coiled-coil domain containing 36–U	*−*3.84	2.43E-06	*−*2.85	6.65E-06
Odf3l1//outer dense fiber of sperm tails 3-like 1–U	*−*3.88	1.54E-05	*−*4.58	1.37E-05
LOC100360582//5',3'-nucleotidase, cytosolic –O	*−*3.89	3.03E-05	*−*2.48	1.69E-04
Dguok//deoxyguanosine kinase –O	*−*3.90	2.09E-05	*−*3.00	5.56E-05
Ick//intestinal cell kinase –D	*−*3.90	7.69E-06	*−*3.30	1.43E-05
Aspscr1//alveolar soft part sarcoma chrom region 1 – O	*−*3.92	4.86E-06	*−*2.01	5.15E-05
Arntl2//aryl hydrocarbon receptor nuclear transl-like 2 – T	*−*3.93	3.45E-06	*−*2.92	8.57E-06
Acy3//aspartoacylase (aminocyclase) 3–O	*−*3.93	3.79E-06	*−*3.47	6.91E-06
Zfp9//zinc finger protein 9–T	*−*3.99	6.67E-06	*−*3.71	1.02E-05
LOC688548//hypothetical protein LOC688548–U	*−*3.99	3.08E-05	*−*3.06	8.11E-05
Fblim1//filamin-binding LIM protein 1–D	*−*4.01	5.05E-06	*−*3.87	7.42E-06
*Kif3c//kinesin family member 3C –O*	*−4.01*	*1.69E-05*	*−3.32*	*3.48E-05*
Gsdmd//gasdermin D –D	*−*4.02	3.08E-06	*−*3.36	6.02E-06
LOC498145//similar to RIKEN cDNA 2810453I06 –U	*−*4.02	2.45E-04	*−*2.89	8.91E-04
*Zfp426l2//zinc finger protein 426-like 2–T*	*−4.02*	*5.00E-05*	*−4.59*	*4.24E-05*
Chst12//carbohydrate (chondroitin 4) sulfotransfer 12– O	*−*4.03	1.13E-05	*−*2.89	3.48E-05
RGD1565316//similar to sphingomyelin phosphodies – U	*−*4.04	7.16E-06	*−*4.18	8.72E-06
Mir143//microRNA mir-143 – T	*−*4.04	4.55E-05	*−*2.67	2.14E-04
Sh3glb2//SH3-domain GRB2-like endophilin B2 – U	*−*4.04	4.68E-05	*−*2.05	8.75E-04
Ccdc8//coiled-coil domain-containing 8 – D	*−*4.06	1.20E-05	*−*2.98	3.48E-05
**Pot1//protection of telomeres 1 homolog (1) – D**	***−*** **4.07**	**5.28E-06**	***−*** **5.21**	**5.01E-06**
*Rbl2//retinoblastoma-like 2 – T*	*−4.07*	*5.76E-06*	*−2.05*	*6.46E-05*
Ankra2//ankyrin repeat, family A (RFXANK-like) 2 – O	*−*4.09	1.26E-05	*−*4.30	1.44E-05
Slc45a4//solute carrier family 45, member 4 – O	*−*4.10	5.05E-06	*−*2.92	1.45E-05
Polm//polymerase (DNA directed) – D	*−*4.13	4.22E-06	*−*4.06	6.03E-06
**Hmbs//hydroxymethylbilane synthase (1) – O**	***−*** **4.16**	**3.51E-06**	***−*** **2.76**	**1.15E-05**
Mrpl40//mitochondrial ribosomal protein L40 – T	*−*4.17	1.45E-04	*−*4.20	1.59E-04
Fastk//Fas-activated serine/threonine kinase – T	*−*4.20	9.96E-06	*−*2.84	3.50E-05
LOC688548//hypothetical protein LOC688548 – U	*−*4.29	1.31E-05	*−*3.27	3.31E-05
LOC691254//hypothetical protein LOC691254 – U	*−*4.39	1.05E-05	*−*4.31	1.37E-05
**Plcb3//phospholipase C, beta 3, PIP-specific (1) – O**	***−*** **4.41**	**2.75E-06**	***−*** **3.20**	**7.06E-06**
Setd6//SET domain-containing 6 – T	*−*4.41	5.99E-06	*−*3.55	1.22E-05
RGD1565222//similar to RIKEN cDNA 4931414P19 – U	*−*4.43	1.17E-05	*−*3.74	2.18E-05
Crot//carnitine O-octanoyltransferase – O	*−*4.44	1.26E-05	*−*3.59	2.66E-05
Haus1//HAUS augmin-like complex, subunit 1 – D	*−*4.46	3.39E-05	*−*4.58	3.85E-05
Paqr7//progestin and adipoQ receptor family memb VII – O	*−*4.52	3.81E-06	*−*5.24	4.54E-06
Slc27a4//solute carrier family 27 (fatty acid transp) – O	*−*4.56	1.11E-05	*−*3.03	3.94E-05
Pde6d//phosphodiesterase 6D, cGMP-specific, rod – O	*−*4.57	5.23E-06	*−*5.82	5.06E-06
Stard3nl//STARD3 N-terminal like – O	*−*4.61	2.60E-06	*−*3.88	5.04E-06
Ssx2ip//synovial sarcoma, X breakpoint 2 interact prot – D	*−*4.64	1.05E-05	*−*3.20	3.31E-05
*Galk1//galactokinase 1 – O*	*−4.68*	*1.03E-05*	*−4.08*	*1.73E-05*
Nipsnap1//nipsnap homolog 1 (C. elegans) – O	*−*4.72	9.38E-06	*−*4.94	1.08E-05
Golph3l//golgi phosphoprotein 3-like – O	*−*4.75	2.90E-06	*−*3.34	7.44E-06
Kprp//keratinocyte proline-rich protein – U	*−*4.79	5.45E-05	*−*5.63	4.47E-05
Nr1h2//nuclear receptor subfamily 1, group H, mem 2 – T	*−*4.94	3.81E-05	*−*4.03	7.47E-05
**Ogg1//8-oxoguanine DNA glycosylase (11) – D**	***−*** **5.11**	**4.60E-06**	***−*** **3.10**	**1.68E-05**
Nsdhl//NAD(P)-dependent steroid dehydrogenase-like – O	*−*5.13	9.08E-07	*−*3.59	2.89E-06
Mir145//microRNA mir-145 – T	*−*5.14	1.50E-06	*−*3.36	4.54E-06
Zfp40//zinc finger protein 40 – T	*−*5.20	7.56E-06	*−*4.07	1.54E-05
RGD1311946//similar to RIKEN cDNA 1810055G02 – U	*−*5.28	1.87E-06	*−*6.39	2.95E-06
Tmem177//transmembrane protein 177 – O	*−*5.41	3.53E-05	*−*7.49	2.30E-05
Rab27a//RAB27A, member RAS oncogene family – T	*−*5.65	3.45E-06	*−*6.40	4.47E-06
Stx2//syntaxin 2 – D	*−*5.82	2.27E-06	*−*4.98	4.54E-06
Stk16//serine/threonine kinase 16 – O	*−*6.35	1.70E-06	*−*5.32	3.58E-06
Ttc30b//tetratricopeptide repeat domain 30B – O	*−*7.12	8.89E-06	*−*7.00	1.13E-05
**Tradd//TNFRSF1A-associated via death domain (5) – T**	***−*** **7.81**	**9.08E-07**	***−*** **7.44**	**1.84E-06**
Zinki//Arg3.1/Arc mRNA-binding zinc finger protein – T	*−*8.80	3.51E-06	*−*7.78	5.65E-06

RVSMC were treated with 3 mM ouabain or K^+^-free medium for 6 hr. Listed are assigned genes whose expression was decreased in K^+^-free medium by more than 3-fold and was different by less than 2-fold in the presence of ouabain compared to K^+^-free medium. GeneChip expression analysis was performed as described in the Methods section. mRNA content in control cells was taken as 1.00. Genes whose expression is altered in ischemia/hypoxia are shown **in bold**. Appearing in parentheses are numbers of citations in PubMed. Given *in italics* are genes whose differential expression was detected in ischemic tissue by whole genome microarray-based analysis [12]–[18]. Data on gene function from GeneCards database (www.genecards.org) were used for identification of gene function. Functional categories: T– regulators of transcription/translation, RNA processing and degradation; D– regulators of cell adhesion, migration, proliferation, differentiation and death; I– inflammation and immune responses; O – others; U – unknown function.

**Table 3:** Hypoxia-responsive transcription factors whose expression was increased in RASMC subjected to Na^+^,K^+^-ATPase inhibition.

Gene symbol Gene title		Fold alterations	
		K^+^-free	ouabain
**HIF**			
**Hif-1b/Arnt**	Aryl hydrocarbon receptor nuclear translocator	1.28	1.24
**Ahr**	Aryl hydrocarbon receptor	1.94	1.34
**NFkB**			
**Ikbkg**	Inhibitor of kappa light polypeptide kinase β	−1.93	−3.06
**Ikbke**	Inhibitor of kappa light polypeptide kinase ε	−2.16	−2.18
**Sike 1**	Suppressor of Ikbke	−5.51	−6.40
**AP-1**			
**Fos**	FBJ osteosarcoma oncogene	7.91	3.95
**Jun**	Jun proto-oncogene	3.57	2.76
**Junb**	Jun B proto-oncogene	3.23	1.29
**Jund**	Jun D proto-oncogene	1.59	1.53
**Atf3**	Activating transcription factor 3	7.83	4.53
**Atf6**	Activating transcription factor 6	1.42	1.36
**Atf1**	Activating transcription factor 1	1.35	1.24
**Maff**	v-maf musculoaponeurotic oncogene homolog F	3.42	2.55
**Mafk**	v-maf musculoaponeurotic oncogene homolog K	3.42	2.75
**CREB**			
**Creb5**	cAMP-responsive element-binding protein 5	1.69	1.36
**Creb1**	cAMP-responsive element-binding protein 1	1.45	1.46
**p53**			
**Mdm2**	Mdm2 p53-binding protein homolog (mouse)	3.85	2.98
**EGR**			
**Egr1**	Early growth response 1	5.15	1.49
**OTHERS**			
**Sp1**	Sp1 transcription factor	2.00	1.50

Na^+^,K^+^-ATPase was inhibited for 6 hr by 3 mM ouabain or K^+^-free medium. Expression of other genes encoding members of HIF-1 (*Hif-1a, Hif-2a, Hif-3a*), NFκB (*p65, cRel, RelB, p50, p52, IκB*) as well as other hypoxia-inducible transcription factors (*p53, Sp3, Gata2, Stat5, Gadd153*) was not significantly changed.

**Figure 5:**
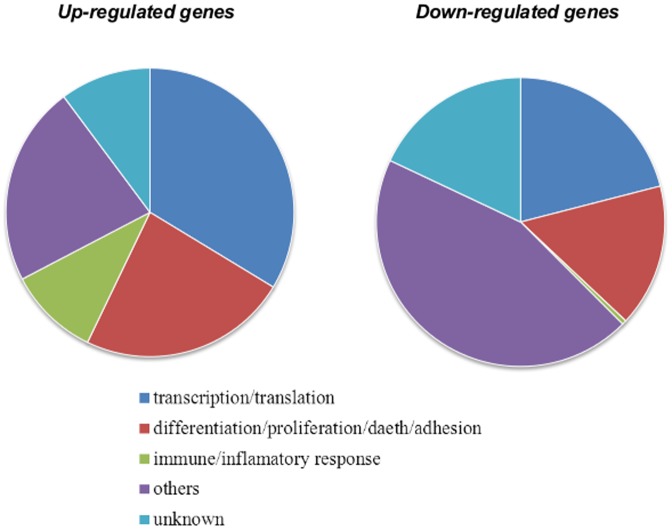
Distribution of up- and down- regulated [Na^+^]_i_/[K^+^]_i_-sensitive genes listed in Tables 2 and 3 among major functional categories.

Although functional characterization is somewhat artificial – because genes are usually multifunctional and fall into several categories – we ascertained that both up- and down-regulated [Na^+^]_i_/[K^+^]_i_-sensitive transcriptomes were enriched with genes involved in transcription/translation, cell adhesion, migration, proliferation, differentiation and death (Tables 1 and 2, Fig. 5). We also noted that, among [Na^+^]_i_/[K^+^]_i_-sensitive genes, the relative content of transcription/translation regulators was ∼3–4-fold higher than in total mammalian genomes [32]. Keeping this in mind, we undertook an additional search for genes encoding HIF-1, AP-1, cyclic AMP response element-binding protein (CREB), nuclear factor kappa-B (NFκB), early growth response factors (EGR), i.e. major transcription factors involved in transcriptomic changes evoked by hypoxia (for review, see [33]). Table 3 demonstrates that Na^+^,K^+^-ATPase inhibition resulted in augmented expression of genes encoding AP-1 and *Egr1* and down-regulation of genes encoding regulators of the NFkB- and p53-mediated signalling pathways. We found less than 2-fold elevation of *Sp1, Creb1* and *Creb5* and lack of any impact of increment of the [Na^+^]_i_/]K^+^]_i_ ratio on the transcription of other hypoxia-inducible transcription factors: *Hif-1a, Hif-1b, Hif-2a, Hif-3a, p65, cRel, RelB, p50, p52, IκB, p53*, *Sp3, Gata2, Stat5, Gadd153*.

### *In silico* search for subset of genes whose expression is affected both elevation of the [Na^+^]_i_/]K^+^]_i_ ratio and hypoxia

To select candidate genes for the investigation of relative impact of HIF-1α- and [Na^+^]_i_/[K^+^]_i_-mediated signaling triggered by hypoxia, we performed a comparative analysis of our results on the identification of [Na^+^]_i_/]K^+^]_i_-sensitive in RASMC and PubMed database for genes affected by hypoxia/ischemia. In Tables 1 and 2, [Na^+^]_i_/]K^+^]_i_-sensitive transcripts found in PubMed database as genes affected by hypoxia and/or ischemia are shown in **bold** with number of publications given in parentheses. Then, we performed and additional search [Na^+^]_i_/]K^+^]_i_- and hypoxia-sensitive genes in manuscripts investigated transcriptomic changes triggered by hypoxia/ischemia using the global gene expression profiling technology [12]-[14]; [16]; [17]. [Na^+^]_i_/[K^+^]_i_-sensitive genes found in these papers are *italicized* in Tables 1 and 2. These two approaches led us to conclusion that relative percentages of hypoxia-sensitive genes among up- and down-regulated [Na^+^]_i_/[K^+^]_i_-sensitive genes were ∼40% and 12%, respectively, i.e. much higher than predicted from random distribution in the rat genome of 280 [Na^+^]_i_/[K^+^]_i_-sensitive genes and 60 hypoxia-sensitive genes annotated in Tables 1 and 2.

### Role of HIF-1α- and [Na^+^]_i_/[K^+^]_i_-mediated signaling

For further investigations, we selected *Cyp1a1, Fos, Atf3, Klf10, Ptgs2, Nr4a1, Per2* and *Hes1*, i.e. genes possessing the highest increments of expression under sustained Na^+^,K^+^-ATPase inhibition and whose implication in the pathogenesis of hypoxia was proved in previous studies. Thus, FOS and ATF3, together with JUN, form dimeric transcription factor AP-1 whose augmented expression was documented in all types of cells subjected to hypoxia [33]. *Ptgs2* encodes an inducible isoform of cyclooxygenase-2 (COX-2) whose role in the pathophysiology of hypoxia is well-documented [34]. *Klf10* is a Kruppel-like zinc-finger transcription factor family member involved in hypoxia-dependent angiogenesis via COX-1 activation [35]. Nerve growth factor IB, also known as *Nur77* or *Nr4a1*, is the nuclear receptor of transcription factors stabilizing HIF-1α, which increases its transcriptional activity [36]. *Hes1* is the basic helix-loop-helix transcription factor whose expression is sharply augmented after ischemic renal failure [37]. The core circadian oscillator is composed of a transcription-translation feedback loop in which *Clock* and *Bmal1* are positive regulators, and *Per1, Per2, Cry1* and *Cry2* act as negative regulators [38]. It has been shown that *Per2* promotes circadian stabilization of HIF-1α activity that is critical for myocardial adaptation to ischemia [39]; [40]. *Cyp1a1* encodes a cytochrome P450 family member and its expression is mediated by HIF-1β [44]. Vascular endothelial growth factor (*Vegfa*) and endothelin (*Edn1*) were chosen as positive controls for canonical HIF-1α-sensitive genes.

**Figure 6:**
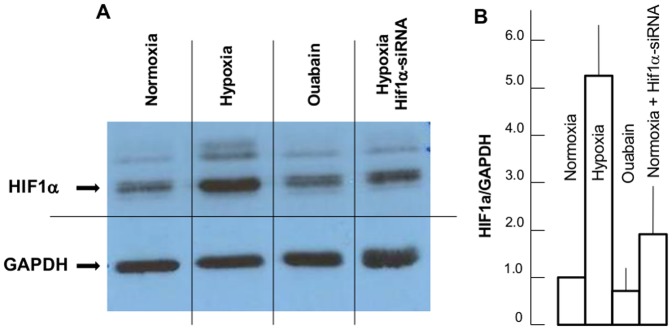
**A**. Representative Western blots of HIF-1α and GAPDH in RASMC subjected to 24-hr incubation under control conditions (normoxia), hypoxia/glucose deprivation, 3 mM ouabain or hypoxia/glucose deprivation in cells transfected with *Hif-1α* siRNA. **B**. Effect of hypoxia/glucose deprivation and ouabain on relative content of HIF-1α protein in RASMC. The HIF-1α/GAPDH ratio in control conditions was taken as 1.00. Data obtained in 3 independent experiments are reported as means ± S.E.

To examine the relative impact of HIF-1α-mediated and [Na^+^]_i_/[K^+^]_i_-dependent signaling, we compared the effects of hypoxia and ouabain on expression of the above-listed selected genes in control high-Na^+^, low-K^+^ medium, after dissipation of the transmembrane gradients of monovalent cations in high-K^+^, low-Na^+^ medium and in cells transfected with *Hif-1a* siRNA. As demonstrated in other cell types studied so far [22]; [41], hypoxia slightly augmented *Hif-1a* mRNA (Table 4) and increased immunoreactive HIF-1α protein content by ∼5-fold (Fig. 6). RASMC transfection with *Hif-1a* siRNA but not with scrambled siRNA decreased *Hif-1a* expression by ∼3-fold and sharply attenuated the rise in HIF-1α protein triggered by hypoxia (Fig. 6). Ouabain increased baseline *Hif-1a* mRNA by ∼50% (Table 4) and slightly curbed HIF-1α protein content (Fig. 6). Confirming previous observations [10], hypoxia increased *Vegfa* and *Edn1* mRNA content by 12- and 4-fold, respectively (Table 5). Transfection with *Hif-1a* siRNA decreased hypoxia-dependent increments of *Vegfa* and *Edn1* mRNA by ∼4- and 2-fold, respectively (Fig. 6). Ouabain did not significantly affect *Vegfa* and augmented *Edn1* mRNA by 2.5-fold. Dissipation of the transmembrane gradients of monovalent cations in low-Na^+^, high-K^+^ medium did not alter the expression of *Vegfa* triggered by hypoxia and decreased *Edn1* mRNA by 2-fold. Viewed collectively, these data strongly support the efficacy of Hif1α-siRNA function.

**Table 4:** Effect of high-K^+^, low-Na^+^ medium and *Hif-1α* siRNA on gene expression triggered by hypoxia and ouabain.

Gene	Cell treatment											
symbol	Control (High-Na^+^, low-K^+^)			Low-Na^+^, high-K^+^			Scrambled siRNA			HIF-1α siRNA		
	*normoxia*	*hypoxia*	*ouabain*	*normoxia*	*hypoxia*	*ouabain*	*normoxia*	*hypoxia*	*ouabain*	*normoxia*	*hypoxia*	*ouabain*
**Hif-1a**	1.00	1.88 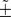 11	1.56 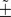 18	0.8 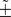 8	1.25 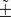 5	1.06 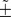 21	0.89 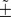 14	1.47 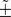 13	1.11 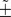 9	0.31 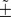 4	0.26 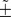 4	0.22 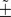 5
**Vegfa**	1.00	12.67 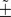 248	1.43 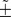 16	1.42 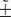 7	14.53 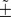 168	1.11 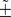 18	0.77 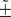 13	11.19 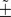 98	1.08 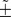 6	0.89 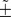 21	3.12 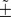 5	1.36 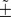 23
**Edn1**	1.00	4.11 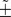 39	2.45 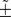 11	1.07 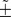 8	2.85 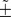 26	0.87 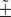 16	1.14 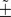 22	4.89 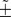 54	2.66 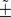 17	1.14 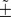 9	1.89 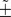 44	2.46 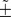 2
**Cypa1**	1.00	0.43 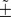 4	8.94 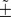 111	1.33 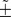 22	0.39 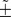 7	1.76 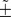 25	0.95 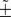 15	0.38 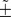 6	10.25 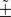 211	0.77 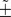 21	0.40 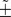 4	13.17 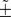 311
**Fos**	1.00	4.12 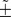 41	5.21 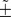 16	1.99 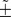 29	2.02 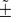 17	1.65 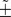 28	0.87 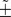 17	5.11 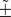 48	5.07 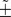 12	1.12 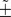 18	4.01 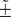 38	4.77 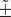 29
**Atf3**	1.00	7.16 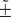 53	5.02 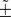 65	2.19 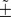 3	3.13 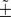 4	2.02 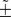 17	1.07 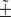 28	6.92 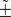 58	5.00 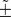 82	0.98 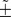 9	3.12 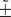 3	5.14 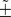 37
**Klf10**	1.00	5.02 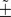 66	4.06 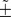 44	1.19 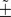 1	2.18 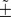 4	1.33 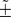 31	1.07 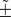 9	3.87 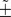 36	4.56 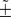 42	1.26 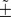 18	3.03 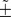 51	3.82 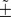 34
**Ptgs2**	1.00	5.14 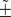 77	9.87 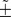 138	2.12 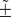 42	1.88 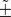 19	1.98 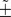 31	1.23 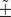 7	6.11 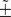 1	9.67 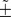 132	0.92 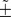 13	4.91 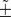 57	9.16 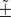 49
**Nr4a1**	1.00	6.11 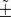 79	6.15 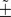 71	1.36 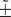 12	2.60 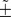 39	1.71 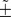 44	0.91 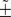 6	3.15 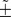 21	7.11 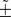 155	0.92 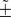 19	2.91 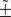 46	7.06 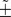 79
**Per2**	1.00	3.02 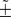 36	6.18 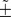 14	0.98 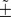 18	1.48 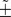 2	1.38 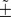 17	1.27 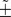 19	3.37 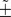 28	5.82 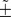 7	1.06 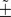 19	3.00 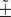 32	7.59 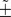 131
**Hes1**	1.00	1.72 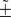 46	3.48 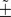 33	1.56 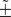 18	2.09 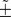 28	1.89 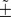 22	0.87 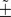 9	1.30 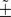 27	3.22 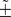 43	1.16 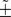 23	1.03 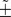 15	4.09 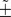 58

Control non-transfected RASMC or RASMC transfected with scrambled or *Hif-1α* siRNA were exposed to hypoxia/glucose deprivation or 3 mM ouabain in control or low-Na^+^, high-K^+^ medium for 24 hr and RNAs content of genes listed in the left column was quantified by real-time quantitative RT-PCR as described in Methods. Means ± S.E. obtained in 3 independent experiments performed in triplicate are shown. Mean values for cells incubated in control medium under normoxic conditions were taken as 1.00.

**Table 5:** Primer sequences for RT-PCR.

Gene symbol	Forward primers	Reverse primers
**Atf3**	TGTCAGTCACCAAGTCTGAGGT	CAGTTTCTCTGACTCCTTCTGC
**Cyp1a1**	ATTTGAGAAGGGCCACATCC	AAACCCAGCTCCAAAGAGGT
**Edn1**	AAAGAACTCCGAGCCCAAAG	CTGATGGCCTCCAACCTTC
**Fos**	GAGCAGCTATCTCCTGAAGAGG	TGATCTGTCTCCGCTTGGA
**Hes1**	CAAACCAAAGACAGCCTCTGA	ATGCCGGGAGCTATCTTTCT
**Hif1a**	AAAGCTCACCTGAGCCTAACA	TGTCCTGAGCTGAAAATGGA
**Klf10**	TCTGTAGCCACCCAGGATGT	GGACAGTTCATCGGAACGAG
**Nr4a1**	GATGCCTCCCCTACCAATCT	GTCACCGGCATCTTCCTTT
**Per2**	GCAGGTGAAGGCTAATGAGG	CACAGCAAACATGTCCGAGT
**Ptgs2**	GGCCATGGAGTGGACTTAAA	TGTCTTTGACTGTGGGAGGA
**Vegfa2**	CATGCGGATCAAACCTCAC	TGGCTTTGTTCTATCTTTCTTTGG

**Figure 7:**
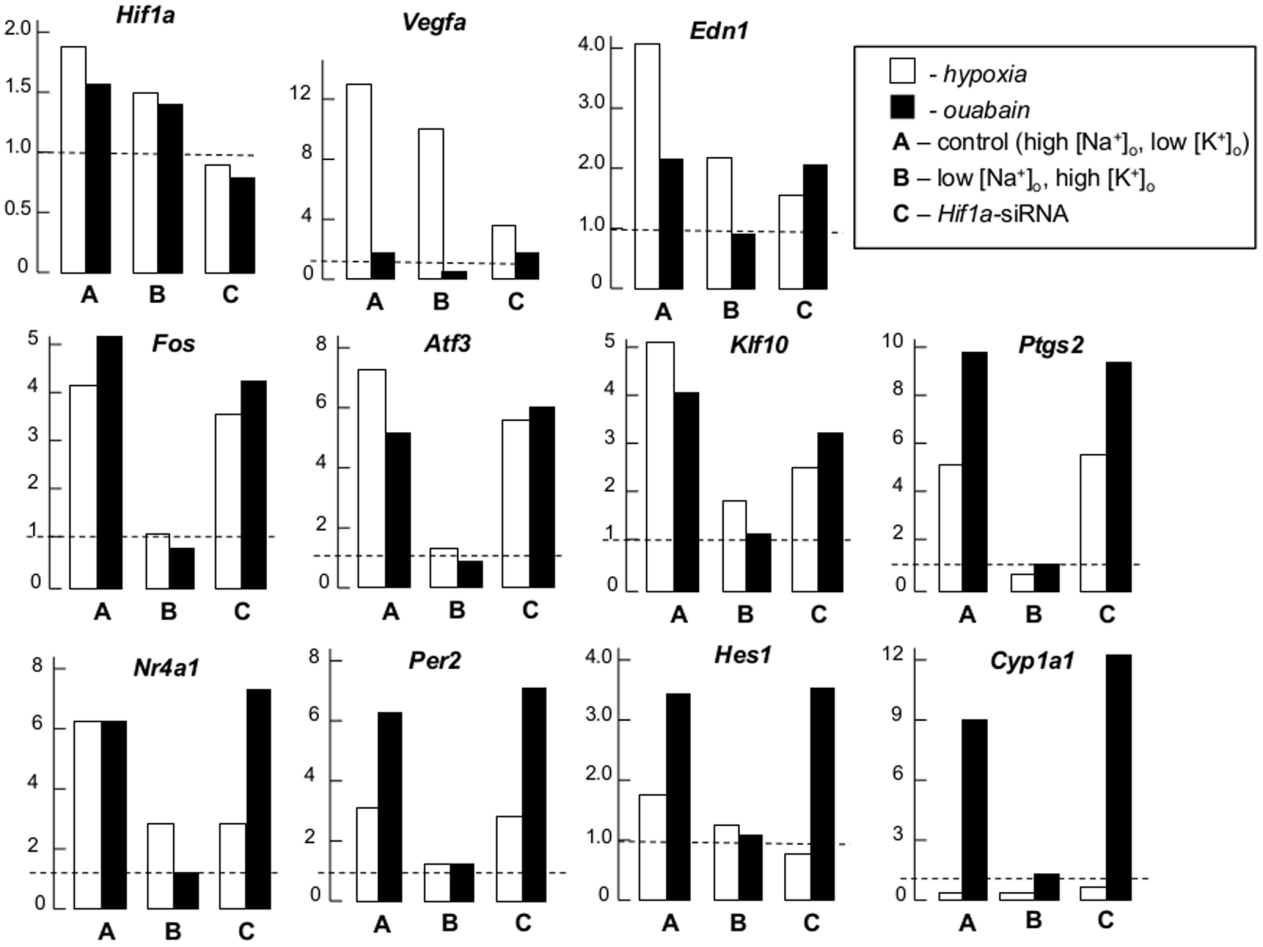
Effect of hypoxia and ouabain on gene expression in RASMC. Cells were exposed to normoxia, hypoxia/glucose deprivation or 3 mM ouabain for 24 hr in control high-Na^+^, low-K^+^ medium (**A, C**), or high-K^+^, low-Na^+^ medium (**B**). In some experiments, RASMC were transfected with *Hif-1α* siRNA (**C**). mRNA content in normoxia was taken as 1.00 and shown by broken lines. For more details, see figure 4 legend.

Dissipation of the transmembrane gradients of monovalent cations completely suppressed increments of *Fos, Atf3, Ptgs2* and *Per2* mRNA and sharply diminished elevations of *Klf10, Edn1, Nr4a1* and *Hes1* expression seen in hypoxic conditions (Fig. 7). Consistent with data obtained in other cell types, including human VSMC [42]; [43], hypoxia increased *Fos, Atf3, Klf10, Ptgs2, Nr4a2, Per2* and *Hes1* expression from 2- to 6-fold (Fig. 7). Transfection with *Hif-1a* siRNA decreased *Klf10* and *Nr4a* mRNA increments evoked by hypoxia by ∼2-fold but did not affect hypoxia-induced *Fos, Atf3, Ptgs2* and *Per2* expression. In contrast to the other genes listed in Table 4, hypoxia decreased *Cyp1a1* mRNA by 2-fold in concordance with attenuated *Cyp1a1* expression in the human microvasculature subjected to hypoxia [44]. The expression of all 8 tested genes was heightened from 3- to 10-fold in the presence of ouabain. These increments were completely abolished under dissipation of the transmembrane gradients of monovalent cations in low-Na^+^, high-K^+^ medium. In contrast to low-Na^+^, high-K^+^ medium, transfection with *Hif-1a* siRNA did not affect the expression of these genes in ouabain-treated RASMC (Table 4). Dissipation of the transmembrane gradient of monovalent cations completely inhibited increments of *Fos, Atf3, Ptgs2* and *Per2* mRNA and sharply diminished elevation of *Klf10, Edn1, Nr4a1* and *Hes1* expression seen in hypoxic conditions (Fig. 7).

### Localization of (A/G)CGTG hypoxia response elements within 5′-UTR

**Figure 8:**
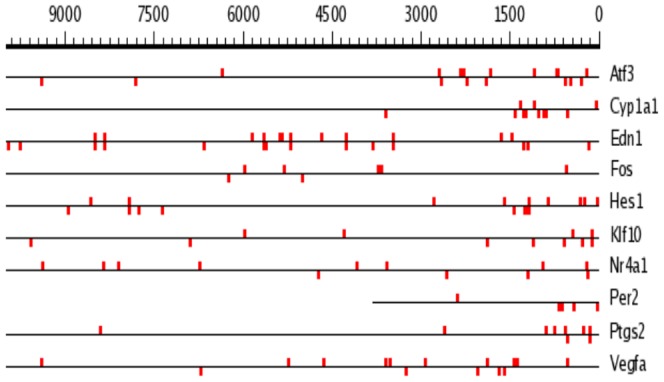
Position of (A/G)CGTG consensus within 10,000 bp 5′-UTR of genes listed in Table 5.

**Figure 9:**
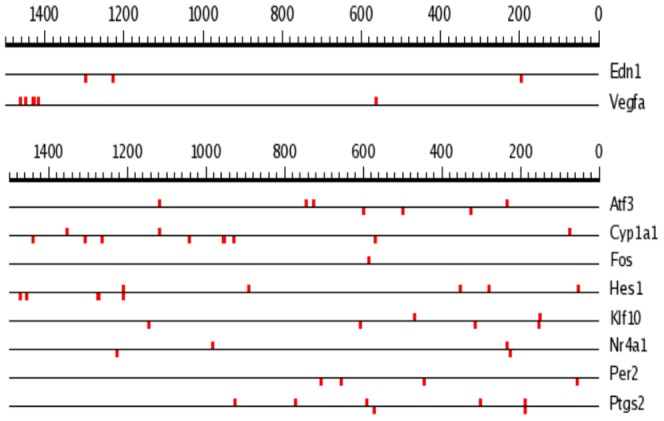
Position of (A/G)CGTG consensus within 1,500 bp 5′-UTR of genes listed in Table 5.

Several research teams reported that HIF-1α regulates gene expression in ischemic tissues via interaction of HIF-1α/HIF-1β heterodimer with HREs containing (A/G)CGTG consensus in promoter/enhancer regions of the target gene's DNA such as VEGFA [45] and EDN1 [46]. Considering this, we employed SCOPE service (Suite for Computational Identification of Promoter Elements): http://genie.dartmouth.edu/scope/ [47] for the search of (A/G)CGTG consensus within 5′-untranslated regions (5′-UTR) of [Na^+^]_i_/[K^+^]_i_-sensitive genes listed in Table 4. Using this approach we found numerous (A/G)CGTG sequences within 5′-UTR encoding canonical HIF-1-sensitve genes (*Edn1 and Vegfa*) as well as all [Na^+^]_i_/[K^+^]_i_-sensitive genes listed in Table 4. Importantly, we failed to find any fixed position for this consensus within 10,000 bp 5′-UTRs of HIF1α-sensitive vs HIF1α-resistant genes (Fig. 8). Moreover, we observed that in several [Na^+^]_i_/[K^+^]_i_-sensitive genes proximal 1,500 bp segments of 5′-UTRs are more abundant with (A/G)CGTG sequence as compared to canonical HIF-sensitive transcripts (Fig. 9). Thus, 1,500 bp 5′-UTRs of *Atf3* and *Edn1* contains 8 and 3 (A/G)CGTG sequences. This observation is also confirmed by Sig Value parameter having a value of 28.4 for 1500 bp 5′-UTRs of genes listed in Table 5. If the search is not restricted to positions of 1500 bp, Sig Value is negative indicating the absence of predictive capabilities for the consensus sequence.

## Discussion

HIF-1α the sole known oxygen sensor, regulates gene expression in ischemic tissues via interaction of HIF-1α/HIF-1β heterodimer with HREs in promoter/enhancer regions of the target gene's DNA [19]–[22]. Our investigation demonstrates, for the first time, that [Na^+^]_i_/[K^+^]_i_-sensitive excitation-transcription coupling contributes to the transcriptomic changes triggered by hypoxia independently of HIF-1α-mediated signaling. Evidences supporting this conclusion are listed below.

*First*, elevation of the [Na^+^]_i_/[K^+^]_i_ ratio evoked by 6-hr inhibition of Na^+^,K^+^-ATPase by ouabain or K^+^-free medium resulted in differential expression of more than 6,000 transcripts (Fig. 3). The list of [Na^+^]_i_/[K^+^]_i_-sensitive genes whose expression changed by more than 3-fold (Tables 1 and 2) is abundant with genes whose differential expression was reported to be affected by hypoxia or ischemia/reperfusion.

*Second*, 24-hr maintenance of RASMC under hypoxic, glucose-depleted conditions resulted in ∼3-fold elevation of [Na^+^]_i_ and 2-fold attenuation of [K^+^]_i_ (Fig. 2). Previously, it was shown that transient ischemia of cardiac myocytes increased [Na^+^]_i_ from 5-8 to 25–40 mM and decreased [K^+^]_i_ by 30% [48]. Augmentation of the [Na^+^]_i_/[K^+^]_i_ ratio observed in hypoxic RASMC is probably caused by attenuation of intracellular ATP content (Fig. 2) that, in turn, leads to partial inhibition of Na^+^,K^+^-ATPase.

*Third*, RASMC transfection with *Hif-1a* siRNA curbed the increment of HIF-1α protein as well as *Vegfa, Edn1, Klf10* and *Nr4a1* mRNA triggered by ischemia (but did not significantly affect the ∼7-, 5-, 4- and 3-fold elevation of *Atf3, Ptgs2, Fos* and *Per2* expression documented in hypoxic cells (Figs. 6 and 7, Table 4).

*Fourth*, unlike *Hif-1a* siRNA, dissipation of the transmembrane gradients of monovalent cations in low-Na^+^, high-K^+^ medium abolished the increase of *Fos, Atf3, Ptgs2* and *Per2* mRNA and sharply decreased *Klf10, Edn1, Nr4a1* and *Hes1* expression evoked by hypoxia. Significantly, low-Na^+^, high-K^+^ medium completely abrogated elevation of the [Na^+^]_i_/[K^+^]_i_ ratio (Fig. 2) as well as increments of the expression of all investigated genes seen in ouabain-treated RASMC (Table 4, Fig. 7).

Viewed collectively our results demonstrate the dominated role of [Na^+^]_i_/[K^+^]_i_-mediated excitation-transcription coupling in overall transcriptomic changes triggered by ischemic conditions. It was shown that in ischemic tissues HIF-1α increases expression of *Vegfa* and *Edn1* via interaction of HIF-1α/HIF-1β heterodimer with hypoxia response elements (HRE) encoding by 5′-UTR (A/G)CGTG consensus [45]; [46]; [49]. We noted that side-by-side with *Hif-1a* siRNA-sensitive *Vegfa* and *Edn1*, 5′-UTRs of *Hif-1a* siRNA-resistant *Atf3, Ptgs2, Fos* and *Per2* are also abundant with (A/G)CGTG sequences (Fig. 8 and 9). Therefore, the presence of (A/G)CGTG consensus within 5′-UTR is not sufficient alone to predict HIF-1-mediated mechanism of gene expression regulation in hypoxic conditions.

Side-by-side with HIF-1α protein accumulation, hypoxia triggers the expression of other transcription factors listed in Table 3 and reviewed by Cummins et al. [33]). Do these transcription factors contribute to [Na^+^]_i_/[K^+^]_i_-mediated transcriptomic changes evoked by hypoxia? We found a negligible impact of Na^+^,K^+^-ATPase inhibition on *Hif-1β* expression and 2-fold elevation of mRNA encoding aryl hydrocarbon receptors for dioxins, benzopyrenes and other environmental pollutions (AhR) (Table 3). It was shown that, in addition to HIF-1α, HIF-1β forms a dimer with AhR [50] that leads to similar expression levels of the P450 isoforms CYP1A1 and CYP1B1 via binding of AhR/HIF-1β complex to the TNGCGTG consensus sequence in xenobiotic-responsive elements [44]; [51]. However, the involvement of this regulatory pathway in the expression of [Na^+^]_i_/[K^+^]_i_-sensitive genes seems unlikely. Indeed, we observed very modest elevation of *Cyp1b1* expression elicited by ouabain and K^+^-free medium (1.41- and 1.54-fold, respectively), in contrast to the ∼15-fold increase of *Cyp1a1* expression (Table 1). We did not find any changes in mRNAs encoding regulatory (*p65, cRel, RelB, p50, p52)* and inhibitory (*IκB*) subunits of NFκB. *Ikbkg* and *Ikbke* encode kinases that phosphorylate IkB, causing its dissociation and activation of NFkB-mediated transcription, whereas SIKE1 interacts with *Ikbke* and inhibits it [52]. Both *Ikbkg/Ikbke* and *Sike1* expression was decreased up to 5-fold by ouabain and K^+^-free medium (Table 3). Thus, the final outcome of elevation of the [Na^+^]_i_/[K^+^]_i_ ratio on the activity of this regulatory pathway remains unknown. MDM2 is a major negative regulator of p53. Elevation of the [Na^+^]_i_/[K^+^]_i_ ratio increased *Mdm2* expression by ∼3-fold (Table 3), suggesting inhibition of p53 transcription rather than activation detected in hypoxia [53]. We observed that 6-hr inhibition of the Na^+^,K^+^-ATPase resulted in 2–3-fold attenuation of mRNAs encoding AMP-activated protein kinase (AMPK) regulatory subunits *Prkag1* and *Prag2* (Table 2). These data suggest that elevation of the [Na^+^]_i_/[K^+^]_i_ ratio attenuates rather than activates AMPK whose augmented activity was detected in hypoxic cells [53]; [54].

**Figure 10:**
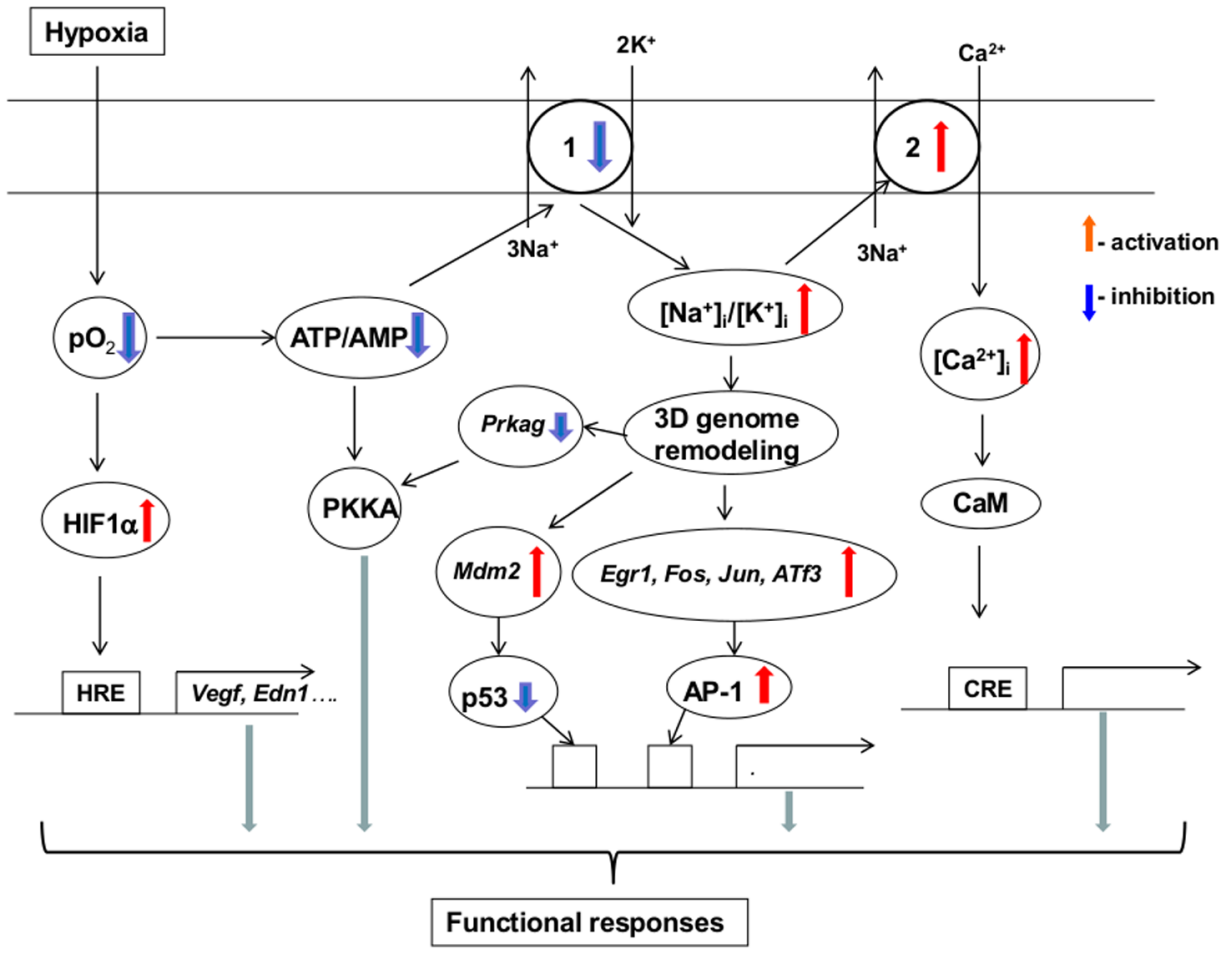
Possible mechanisms of the involvement of elevated [Na^+^]_i_/[K^+^]_i_ ratio in the transcriptomic changes evoked by hypoxia: *a working hypothesis*. 1– Na^+^,K^+^-ATPase; 2– Na^+^/Ca^2+^ exchanger; CaM – calmodulin; CRE – Ca^2+^-response elements. For other abbreviations, see text.

Previously, we demonstrated that 3-hr inhibition of Na^+^,K^+^-ATPase in RASMC by ouabain and K^+^-free medium augmented *Egr1* by ∼5 and 7-fold, respectively [26]. Prolongation of incubation time up to 6 hr decreased the increments of *Egr1* mRNA (Table 3), suggesting transient activation of this transcription factor. We also documented activation of the transcription factor AP-1, indicated by up to 8-fold augmentation of mRNAs encoding its major subunits, including *Fos, Jun, Atf3, Maff and Mafk* (Table 3). These data show that AP-1 and Egr1 are major hypoxia-inducible transcriptions factors that are also activated by elevation of the [Na^+^]_i_/[K^+^]_i_ ratio. Yan and co-workers reported that hypoxia triggered Egr1 in cultured hepatoma-derived cells deficient in HIF-1β [55]. We state here that Fos mRNA accumulation triggered by ouabain occurs in HIF-1α-deficient RASMC (Table 4), indicating that that both the Egr1 and AP-1 pathways are initiated in response to oxygen deprivation independently of HIF-1. As an alternative hypothesis, we propose that activation of *Egr1*, AP-1 and other *Hif-1α* siRNA-resistant genes listed in Table 4 in hypoxic cells is mediated by ATP depletion, Na^+^,K^+^-ATPase inhibition and dissipation of the transmembrane gradients of monovalent cations (Fig. 10).

It was shown that gain of Na^+^_i_ rather than loss of K^+^_i_ sparks augmented *Fos* expression in RASMC [56]. Numerous studies have disclosed that [Na^+^]_i_ elevation heightens [Ca^2+^]_i_ via activation of Na^+^_i_/Ca^2+^_o_ exchanger as well as via depolarization and activation of voltage-gated Ca^2+^ channels (for review, see [57]; [58]). It has been well-documented that [Ca^2+^]_i_ elevation affects gene expression by activation of cAMP-response elements via CREB phosphorylation by (Ca^2+^+calmodulin)-dependent protein kinase and nuclear factor AT (NFAT) dephosphorylation by calcineurin [59]. In previous investigation, we found that addition of extracellular (EGTA and intracellular (BAPTA) Ca2+ chelators increased rather than decreased the number of [Na^+^]i/[K^+^]i-sensitive genes [26]. It should be noted, however, that these compounds may affects cellular functions independently of Ca^2+^ depletion. Thus, we observed that addition of EGTA increases permeability of vascular smooth muscle cells for Na^+^ [60]. Thus, additional experiments should be performed to clarify relative impact of Ca^2+^-mediated and -independent signaling shown in Fig. 10 in transcriptomics changes evoked by hypoxia.

## Conclusion

We report here that elevation of the [Na^+^]_i_/[K^+^]_i_ ratio contributes to transcriptomic changes triggered by hypoxia and glucose deprivation independently of HIF-1—mediated signalling. The molecular origin of the upstream sensor and the relative contribution of the gain of [Na^+^]_i_ and loss of [K^+^]_i_ in the triggering of this novel signalling pathway, including the augmented expression of hypoxia-inducible Egr1 and AP-1 transcription factors, remains unknown. Recent studies have demonstrated that modulation of histone methylation via an epigenetic mechanism is a key device that cells use to adapt to hypoxia [61]. Increasing evidence indicates that side-by-side with regulation of the 5′-UTR by transcription factors, gene activation or silencing is under the complex control of 3-dimensional positioning of genetic materials and chromatin in nuclear spaces [62]; [63]. The role of the [Na^+^]_i_/[K^+^]_i_ ratio in the epigenetic regulation of 3-dimensional genome organization and its relationship to gene silencing and activation are currently being examined in our laboratory.

## Methods

### Cell culture

Rat aortic smooth muscle cells (RASMC), purchased from Lonza (Walkersville, MD, USA), were grown at 37°C in a CO_2_ incubator in Dulbecco's modified Eagle medium (DMEM, Invitrogen, Carlsbad, CA, USA) supplemented with 10% fetal bovine serum (FBS), 100 U/ml penicillin, 100 µg/ml streptomycin, and subjected to less than 10 passages. To establish quiescence, the cells were incubated for 24 hr in media in which FBS concentration was reduced to 0.2%.

### Cell treatment

Quiescent cells were washed with K^+^-free DMEM (Sp-DMEM; Invitrogen) and incubated for 6 hr at 37°C in a humidified atmosphere with 5% CO_2_/balance air in control DMEM, K^+^-free DMEM and DMEM containing 3 mM ouabain. In some experiments, we used DMEM-like medium containing (in mM) NaCl 109.4; KCl 5.4; CaCl_2_ 1.8; MgSO_4_ 0.8; NaHCO_3_ 29.8; NaH_2_PO_4_ 0.9; HEPES 8.4; glucose 5, vitamins and amino acids at concentrations indicated for DMEM recipes ([Na^+^]_o_ = 140.1 mM; [K^+^]_o_ = 5.4) or low-Na^+^, high-K^+^ medium where NaCl was substituted by KCl ([Na^+^]_o_ = 30.7 mM; [K^+^]_o_ = 114.8). Previously, we reported that [K^+^]_o_ elevation caused RASMC depolarization that, in turn, increased Fos expression via activation of L-type voltage-gated Ca^2+^ channels [56]. To inhibit this signalling pathway, 0.1 µM nicardipine was added in certain experiments. To trigger hypoxia, RASMC were incubated in custom-designed, air-tight, flow-through cuvettes in media containing 0.5 mM glucose under substitution of 5% CO_2_/air by 5% CO_2_/N_2_. Eight hr after incubation in a CO_2_/N_2_ environment, pO2 was ∼30 mm Hg compared to ∼150 mmHg in normoxia. We did not observe any impact of these treatment protocols on RASMC survival, estimated by lactate dehydrogenase release, caspase-3 activity and chromatin cleavage (data not included).

### Intracellular content of monovalent ions

Intracellular K^+^, Na^+^ and Cl^-^ content was measured as the steady-state distribution of extra- and intracellular ^86^Rb, ^22^Na and ^36^Cl, respectively. To establish isotope equilibrium, cells growing in 12-well plates were preincubated for 3 hr in control or K^+^-free medium (Sp-DMEM+Ca) containing 0.5 µCi/ml ^86^RbCl, 4 µCi/ml ^22^NaCl or 3 H^36^Cl with ouabain added for the next 3 hr. To test the action of K^+^-free medium, the cells were washed twice with ice-cold Sp-DMEM+Ca. Then, cells loaded with ^22^Na or ^36^Cl were transferred to Sp-DMEM+Ca containing ^22^NaCl and H^36^Cl, respectively, whereas cells loaded with ^86^Rb were transferred to isotope-free Sp-DMEM+Ca. After 3 hr, they were transferred onto ice, washed 4 times with 2 ml of ice-cold medium W containing 100 mM MgCl_2_ and 10 mM HEPES-tris buffer (pH 7.4). The washing medium was aspirated and the cells lysed with 1% SDS and 4 mM EDTA solution. Radioactivity of the incubation media and cell lysates was quantified, and intracellular cation content was calculated as *A/am*, where *A* was the radioactivity of the samples (cpm), *a* was the specific radioactivity of ^86^Rb (K^+^), ^22^Na or ^36^Cl in medium (cpm/nmol), and *m* was protein content (mg). For more details, see [64].

### Intracellular ATP content

Intracellular ATP content was measured by assaying luciferase-dependent luminescence with ATP bioluminescent assay kit (Sigma, St. Louis, MO, USA), as described in detail elsewhere [65].

### Intracellula Na^+^, K^+^, Cl^-^ and ATP concentrations

Intracellular Na^+^, K^+^, Cl^-^ and ATP concentrations were calculated on the basis of intracellular water volume in cells seeded in 12-well plates. The volume of intracellular water was measured as [^14^C]-urea available space and calculated as *V = A_c_/A_m_m*, where *A_c_* was radioactivity of the cells after 30-min incubation with 2 µCi/ml [^14^C]-urea (dpm), *A_m_* was radioactivity of the incubation medium (dmp/µl), and *m* was protein content in cell lysates (mg) [66].

### Transfection

Transfection of HIF-1α si-RNA (5′-AGAGGUGGAUAUGUCUGGG-3′) and scrambled siRNA (5′-AGGAUGUGACGGAUUGUGGTT-3′) was undertaken in the presence of Oligofectamine 228 (Invitrogen), as suggested by the manufacturer and described in detail elsewhere [67]. Afterwards, the cells were incubated for 24 hr under control and hypoxic conditions, as indicated above.

### RNA isolatio

Total RNA was extracted from cells grown in 6-well plates with TRIzol reagent (Invitrogen) and purified with RNeasy MinElute cleanup kit (Qiagen, Valencia, CA, USA), following the manufacturers' protocols. Only RNA samples that had more than 7.0 RNA integrity number and no detectable genomic DNA contamination were considered for subsequent gene array analyses. RNA quality was assessed by 2100 Bioanalyzer (Agilent Technologies, Palo Alto, CA, USA). Microarray experiments were performed with GeneChip Human Gene 1.0 ST array (which detects 28,869 gene products) and GeneChip Rat Gene 1.0 ST array (which detects 27,342 gene products). On both arrays, each gene was represented by approximately 26 probes along the entire transcript's length (Affymetrix, Santa Clara, CA, USA). 100 ng of total RNA from each sample were processed with Ambion WT expression kit (Invitrogen), a reverse transcription method that specifically primes non-ribosomal RNA, including both poly(A) and non-poly(A) mRNA, and generates sense-strand cDNA as final product. 5.5 µg of single-stranded cDNA was fragmented and labeled by Affymetrix GeneChip WT terminal labeling kit, with 2.0 µg of the resulting cDNA hybridized on chips.

### GeneChip expression analysis

RNA samples obtained from control cells and cells subjected to Na^+^,K^+^-ATPase inhibition with ouabain and K^+^-free medium in 3 independent experiments were employed for GeneChip expression analysis. The controls and treatments were performed in parallel in each experiment independently from the other experiments. The entire hybridization procedure was conducted with the Affymetrix GeneChip system according to the manufacturer's recommended protocol. The hybridization results were evaluated with Affymetrix GeneChip Command Console Software. Chip quality was assessed by Affymetrix Expression Console. The data were analyzed by Partek Genomics Suite (Partek, St. Louis, MO, USA) and uploaded on the GEO repository with the accession number http://www.ncbi.nlm.nih.gov/geo/query/acc.cgi?acc=GSE61131.

The normalized data then underwent principal component analysis (PCA) [27] to identify patterns in the dataset and highlight similarities and differences among samples. Major sources of variability found within the dataset by PCA served as grouping variabilities for analysis of variance with n = 4 for each group of samples. The ensuing data were filtered to identify transcripts with statistically significant variations of expression among groups that were modulated by at least 20%, with multiple testing corrections by the false discovery rate. Calculated *p*-values and geometric fold changes for each probe set identifier were imported into Ingenuity Pathway Analysis (Ingenuity Systems, http://www.ingenuity.com) to ascertain networks, biological functions and their pathophysiological implications. Functional information on regulated genes was also obtained from publications in PubMed.

### Real-time quantitative reverse transcription-polymerase chain reaction (RT-PCR)

RT-PCR was performed with Express SYBR GreenER qPCR Supermix kit (Invitrogen) according to the manufacturer's instructions. The reaction was carried out with a 7900 HT Fast RT – PCR system (Applied Biosystems, Foster City, CA, USA). The primers presented in Table 5 were designed with Primer3Plus online software from consensus sequences provided by Affymetrix for each gene of interest. All experiments were analyzed in duplicate. β_2_ microglobulin mRNA expression served to normalize and compare the expression values of genes of interest. The results were quantified by the ΔΔCt method in Microsoft Excel.

### Western blotting

RASMC seeded in 12-well plates were lysed on ice in 0.125 ml of buffer containing 150 mM NaCl, 1% Triton X-100, 0.1% SDS, 2 mM EDTA, 2 mM EGTA, 25 mM HEPES (pH 7.5), 10% glycerol, 1 mM NaF, 200 µM Na_3_VO_4_, and protease inhibitors (1 µg/ml leupeptin, 1 µg/ml aprotinin and 1 mM PMSF). The lysates were cleared off insoluble material by centrifugation at 20,000xg for 10 min, treated for 5 min at 95°C, and subjected to sodium dodecyl sulphate-polyacrylamide gel electrophoresis with 4% and 10% polyacrylamide in stacking and resolving gels, respectively. Proteins were transferred to Immobilon-P nitrocellulose membranes (Amersham, Mississauga, ON, Canada), blocked for 1 h at room temperature with 5% dry, fat-free milk dissolved in PBS and incubated overnight at 4°C with the antibodies listed below. The membranes were then treated with horseradish peroxide-conjugated secondary antibodies and developed by enhanced chemiluminescence reaction (Amersham) in accordance with the manufacturer's instructions. Digital chemiluminescence images were taken and quantified by LAS-3000 luminescent analyzer (Fujifilm, Japan).

### Chemicals

Methyl-[^3^H]-thymidine was purchased from ICN Biomedicals, Inc. (Irvine, CA, USA). ^22^NaCl, ^86^RbCl, H^36^Cl and [^14^C]-urea were obtained from PerkinElmer (Waltham, MA, USA), Isotope (St. Petersburg, Russia) and Amersham (Montreal, QC, Canada). DEVD-AMC, DEVD-CHO and z-VAD.fmk were procured from BIOMOL Research Laboratories (Plymouth Meeting, PA, USA). Anti-HIF-1α and anti-GAPDH antibodies were sourced from Merck Millipore (Billerica, MA, USA). The remaining chemicals were supplied by Gibco BRL (Gaithersburg, MO, USA), Calbiochem (La Jolla, CA, USA), Sigma and Anachemia (Montreal, QC, Canada).

### Data analysis

The main program for transcriptomic data analysis was Agilent's Genespring 7.0. Probe set intensity levels were extracted from scanned arrays by the Affymetrix GeneChip Operating Software (version 1.2) and normalized (MAS5) by all probe sets. The probe sets were then filtered on flags (present, marginal, or absent), and expression levels were quantified. Statistically significant probe sets were identified by the false discovery rate followed by 2-way ANOVA with strain and age as major factors. Student's t-test was used for 2-group comparisons. When comparing more than 2 groups, 2-way ANOVA was employed with strain and age as the main factors, followed by Tukey's honest significant difference post-hoc test. Correlation analyses were performed with Pearson product-moment correlation coefficient (r). Null hypothesis was rejected whenever p<0.05.
